# Deep-sea *in situ* and laboratory multi-omics provide insights into the sulfur assimilation of a deep-sea *Chloroflexota* bacterium

**DOI:** 10.1128/mbio.00004-24

**Published:** 2024-02-28

**Authors:** Rikuan Zheng, Chong Wang, Chaomin Sun

**Affiliations:** 1 CAS and Shandong Province Key Laboratory of Experimental Marine Biology & Center of Deep Sea Research, Institute of Oceanology, Chinese Academy of Sciences, Qingdao, China; 2 Laboratory for Marine Biology and Biotechnology, Qingdao Marine Science and Technology Center, Qingdao, China; 3 Center of Ocean Mega-Science, Chinese Academy of Sciences, Qingdao, China; 4 College of Earth Science, University of Chinese Academy of Sciences, Beijing, China; University of Vienna, Vienna, Austria

**Keywords:** deep-sea *Chloroflexota*, sulfur assimilation, proteomics, *in situ *transcriptomics, metagenomics, metatranscriptomics

## Abstract

*Chloroflexota* bacteria are abundant and globally distributed in various deep-sea ecosystems. It has been reported based on metagenomics data that two deep-sea *Chloroflexota* lineages (the SAR202 group and *Dehalococcoidia* class) have the potential to drive sulfur cycling. However, the absence of cultured *Chloroflexota* representatives is a significant bottleneck toward understanding their contribution to the deep-sea sulfur cycling. In this study, we find that *Phototrophicus methaneseepsis* ZRK33 isolated from deep-sea sediment has a heterotrophic lifestyle and can assimilate sulfate and thiosulfate. Using combined physiological, genomic, proteomic, and *in situ* transcriptomic methods, we find that strain ZRK33 can perform assimilatory sulfate reduction in both laboratory and deep-sea conditions. Metabolism of sulfate or thiosulfate by strain ZRK33 significantly promotes the transport and degradation of various macromolecules and thereby stimulates the energy production. In addition, metagenomic results show that genes associated with assimilatory and dissimilatory sulfate reduction are ubiquitously distributed in the metagenome-assembled genomes of *Chloroflexota* members derived from deep-sea sediments. Metatranscriptomic results also show that the expression levels of related genes are upregulated, strongly suggesting that *Chloroflexota* bacteria may play undocumented roles in deep-sea sulfur cycling.

**IMPORTANCE:**

The cycling of sulfur is one of Earth's major biogeochemical processes and is closely related to the energy metabolism of microorganisms living in the deep-sea cold seep and hydrothermal vents. To date, some of the members of *Chloroflexota* are proposed to play a previously unrecognized role in sulfur cycling. However, the sulfur metabolic characteristics of deep-sea *Chloroflexota* bacteria have never been reported, and remain to be verified in cultured deep-sea representatives. Here, we show that the deep-sea *Chloroflexota* bacterium ZRK33 can perform sulfate assimilation in both laboratory and deep-sea conditions, which expands our knowledge of the sulfur metabolic potential of deep-sea *Chloroflexota* bacteria. We also show that the genes associated with assimilatory and dissimilatory sulfate reduction ubiquitously distribute in the deep-sea *Chloroflexota* members, providing hints to the roles of *Chloroflexota* bacteria in deep-sea sulfur biogeochemical cycling.

## INTRODUCTION

The deep marine subsurface is one of the least-understood habitats on Earth and is estimated to contain up to 3 × 10^29^ microbial cells, which is equivalent to the combined microbial biomass of the oceanic water column and terrestrial soil (1). The prokaryotic biomass in deep marine subsurface sediments exceeds 10^5^ microbial cells/cm^3^ even at depths of nearly 1,000 m below the seafloor (2, 3). These microorganisms are the primary drivers of elemental cycles within deep marine subsurface sediments and play key roles in the recycling of biogeochemical nutrients to the water column (4). Members of the phylum *Chloroflexota* are widely distributed in various environments with high abundance. For example, the number of *Chloroflexota* bacteria is equivalent to other total bacterial counts in some marine subsurface sediments (3, 5–8). Therefore, the phylum *Chloroflexota* is the essential group for maintaining the population equilibrium of marine subsurface ecosystems (9–12). Concomitant with an expansion of the known *Chloroflexota* phylum from the utilization of cultivation-independent techniques has been the remarkable diversity of as-yet uncultivated *Chloroflexota* bacteria (13), indicating that immeasurable novel lineages of *Chloroflexota* exist in nature. Despite *Chloroflexota* bacteria being among the first widespread microbial lineages discovered in deep-sea environments (14), we still lack cultured representatives (especially those with a relatively fast growth rate) and their physiological, and ecological properties are still obscure (15–17). For example, until now, only basic physiological characteristics of two cultured strains of *Chloroflexota* from deep-sea sediments are available (16, 17), and both strains have extremely slow growth rates (doubling time from 1.5 to 19 days). Moreover, their central metabolism and contribution to biogeochemical processes, including sulfur cycling, are largely unknown.

The cycling of sulfur is one of Earth’s major biogeochemical processes and is closely related to the energy metabolism of microorganisms living in the cold seep and hydrothermal vents (18–20). Importantly, coupling of sulfate/sulfite reduction to oxidation of H_2_, small chain fatty acids, or other carbon compounds limits the availability of these substrates to other microorganisms, such as methanogens, and alters the energetics via syntrophic interactions that affect the methane production (18). Given the importance of sulfur cycling in deep biospheres, it is vital that we understand which organisms can carry out the reactions and pathways involved (20). Based on metagenomic sequencing results, some SAR202 members of the phylum of *Chloroflexota* are predicted to be sulfite-oxidizers, making them potential key players in the sulfur cycle of the deep marine environment (21). Based on single-cell genomic sequencing results, some *Dehalococcoidia* members have been demonstrated to possess diverse genes encoding dissimilatory sulfite reductase (4), suggesting that *Dehalococcoidia* bacteria could drive sulfite reduction and respire oxidized sulfur compounds. Taken together, some *Chloroflexota* members are believed to play a previously unrecognized role in sulfur cycling, but the sulfur metabolic characteristics of deep-sea *Chloroflexota* bacteria have never been reported.

In our previous study, we isolated a novel member of *Chloroflexota*, *Phototrophicus methaneseepsis* ZRK33, from the deep-sea sediment, and revealed its phototrophic lifestyle (22). However, its contribution to biogeochemical cycling is still unclear. In this study, we found that organic nutrients, sulfate, and thiosulfate could promote *P. methaneseepsis* ZRK33 growth. Combining physiological, proteomic, and *in situ* transcriptomic approaches, we confirmed the presence of assimilatory sulfate reduction in strain ZRK33 in both laboratory and deep-sea conditions. Finally, we also reveal that genes encoding key enzymes driving both assimilatory and dissimilatory sulfate reduction are broadly distributed and upregulated in deep-sea *Chloroflexota* bacteria.

## RESULTS AND DISCUSSION

### Organic nutrients promote *P*. *methaneseepsis* ZRK33 growth

**Fig 1:**
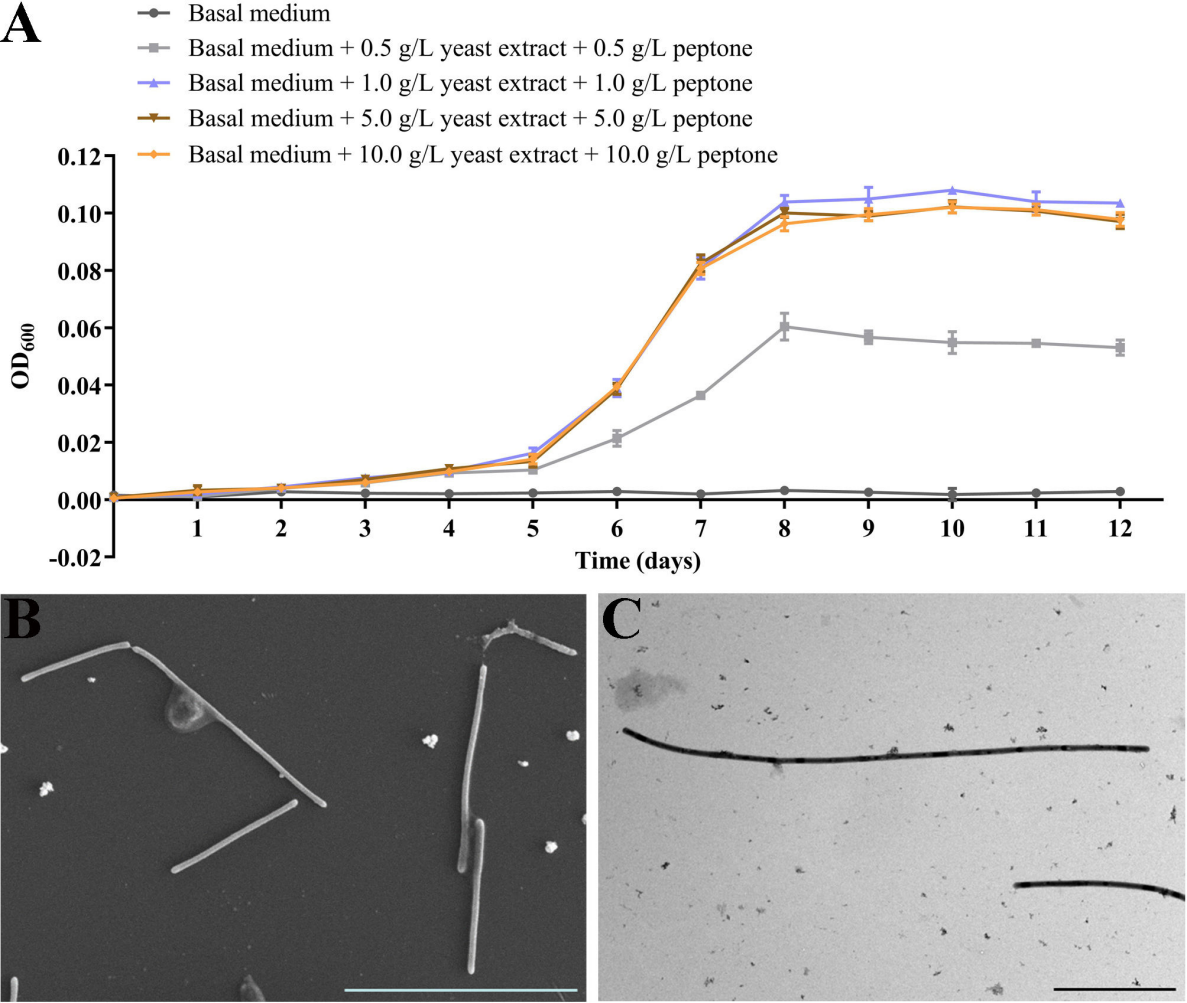
Growth assay and morphology of *P. methaneseepsis* ZRK33 strains cultivated in basal medium or basal medium supplemented with different concentrations of yeast extract and peptone. (**A**) Growth curves of ZRK33 strains cultivated in different media. (**B**) SEM observation of ZRK33 strains cultivated in rich medium. (**C**) TEM observation of ZRK33 strains cultivated in rich medium. The bar is 10 µm in panels (B and C).

Based on previous metagenomic analysis, many *Chloroflexota* bacteria have heterotrophic lifestyles with the potential to degrade a wide range of organic carbon compounds (23). However, the true metabolic traits of deep-sea *Chloroflexota* bacteria are still unclear. We therefore selected a deep-sea *Chloroflexota* representative (*P. methaneseepsis* ZRK33) that we isolated previously and investigated its physiological characteristics. Growth assay results showed that strain ZRK33 grew at a very low rate in basal medium (Fig. 1A), while the supplement of organic nutrients (containing 0.5–10 g/L yeast extract and 0.5–10 g/L peptone) could effectively promote its growth. Specifically, strain ZRK33 grew at a similar rate when cultured in basal medium plus 1.0 g/L, 5.0 g/L, or 10.0 g/L yeast extract and same amount of peptone, and the growth rate was two times that in basal medium supplemented with 0.5 g/L yeast extract and 0.5 g/L peptone (Fig. 1A). Thus, we decided to add 1.0 g/L yeast extract and 1.0 g/L peptone to the basal medium as a rich medium for future culture of strain ZRK33. Cells of strain ZRK33 cultivated in a rich medium are filamentous, generally more than 10 µm long and 0.5–0.6 µm wide under scanning electron microscope (SEM) and transmission electron microscope (TEM) observation (Fig. 1B and C), which is similar to the morphology of two other deep-sea *Chloroflexota* bacteria (16, 17) that required yeast extract for growth. These results suggest that yeast extract is necessary for the growth of deep-sea *Chloroflexota* bacteria, indicating they might need rich nutrients for better growth regardless of their harsh living conditions.

### *P*. *methaneseepsis* ZRK33 assimilates sulfate and thiosulfate

**Fig 2:**
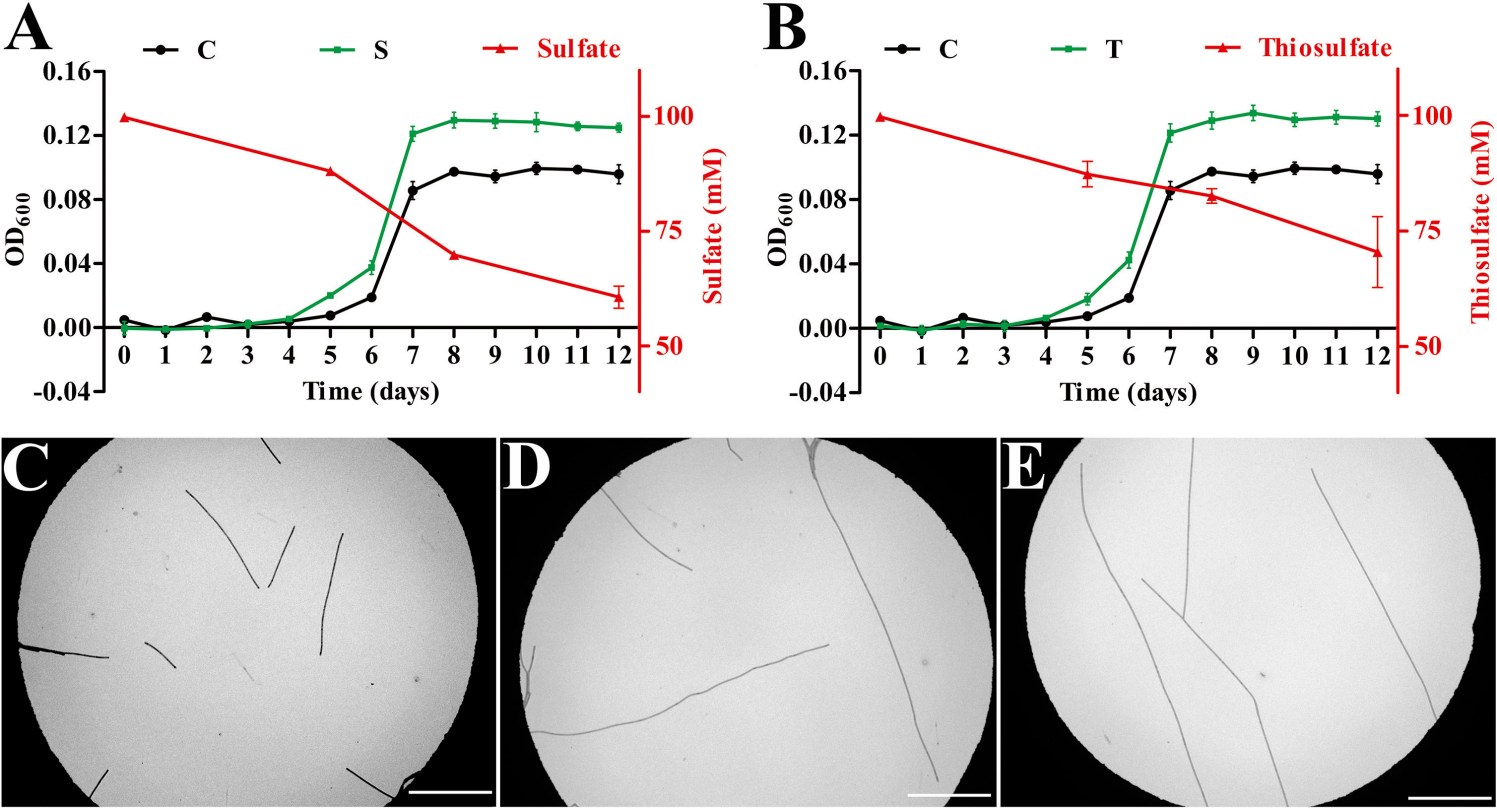
Sulfate assimilation of *P. methaneseepsis* ZRK33. (**A**) Growth assay and sulfate metabolization of strain ZRK33 cultured in rich medium alone or supplemented with 100 mM Na_2_SO_4_. (**B**) Growth assay and thiosulfate metabolism of strain ZRK33 cultured in rich medium alone or supplemented with 100 mM Na_2_S_2_O_3_. “C” indicates the control group, where strain ZRK33 was cultured in rich medium alone; “S” indicates the sulfate-treated group, where strain ZRK33 was cultured in rich medium supplemented with 100 mM Na_2_SO_4_; “T” indicates the thiosulfate-treated group, where strain ZRK33 was cultured in rich medium supplemented with 100 mM Na_2_S_2_O_3_. The black lines represent the growth curves of the control group; the green lines represent the growth curves of experimental groups; the red lines represent the effects of varying concentrations of Na_2_SO_4_ or Na_2_S_2_O_3_. TEM observation of strain ZRK33 cultured in rich medium (**C**), rich medium supplemented with 100 mM Na_2_SO_4_ (**D**), and rich medium supplemented with 100 mM Na_2_S_2_O_3_ (**E**). The bar is 20 µm in panels (C–E).

Cycling of sulfur is a dominant metabolic pathway for marine subsurface microorganisms (19, 24), and deep-sea *Chloroflexota* bacteria were predicted to respire oxidized sulfur compounds (4) and metabolize multiple organosulfur compounds (21) based on metagenomics data. However, to date, no studies based on pure cultures have verified that deep-sea *Chloroflexota* members can perform sulfur metabolism, due to a lack of cultivated representatives for dominant deep-sea lineages. We analyzed the genome of strain ZRK33 and found that it had a set of genes for assimilatory sulfate reduction (Fig. S1; Table S1). Indeed, strain ZRK33 was isolated from the deep-sea cold seep where there is a rich variety of different sulfur-containing compounds (18, 19). Subsequently, we tested the effects of different sulfur-containing inorganic substances (including Na_2_SO_4_, Na_2_SO_3_, Na_2_S_2_O_3_, and Na_2_S) on the growth of strain ZRK33. We found that supplementation of high concentrations (100 mM) of Na_2_SO_4_ and Na_2_S_2_O_3_ could significantly promote the growth of strain ZRK33 (Fig. 2A and B). However, low concentrations of Na_2_SO_4_ and Na_2_S_2_O_3_ (20 mM) had no evident effect on the growth of strain ZRK33 (Fig. S2A and B), indicating this bacterium prefers to utilize high concentrations of Na_2_SO_4_ and Na_2_S_2_O_3_. Meanwhile, concentrations of Na_2_SO_4_ and Na_2_S_2_O_3_ were decreased from 100 mM to 60 mM and 70 mM, respectively, with growth of strain ZRK33 for 12 days, suggesting that strain ZRK33 can effectively metabolize Na_2_SO_4_ and Na_2_S_2_O_3_ (Fig. 2A and B). Moreover, strain ZRK33 average cell length became longer in rich medium supplemented with Na_2_SO_4_ (Fig. 2D) or Na_2_S_2_O_3_ (Fig. 2E) compared to rich medium alone (Fig. 2C), strongly suggesting that ZRK33 could assimilate Na_2_SO_4_ and Na_2_S_2_O_3_ to form organic sulfides. In comparison, supplementation of a very low concentration (1 mM) of Na_2_SO_3_ and Na_2_S inhibited the growth of strain ZRK33 (Fig. S2C and D), indicating that SO_3_^2−^ and S^2−^ were harmful for strain ZRK33 growth, which could also inhibit the growth of other bacteria (15, 25). Given the high concentrations of different sulfur-containing compounds in the cold seep and the ability of some microbes to enrich sulfur-containing compounds [such as elemental sulfur and polysulfide (19, 26)], we therefore suggest that strain ZRK33 may metabolize some sulfur-containing compounds in the deep sea.

### Proteomic analyses of sulfate assimilation in *P*. *methaneseepsis* ZRK33

**Fig 3:**
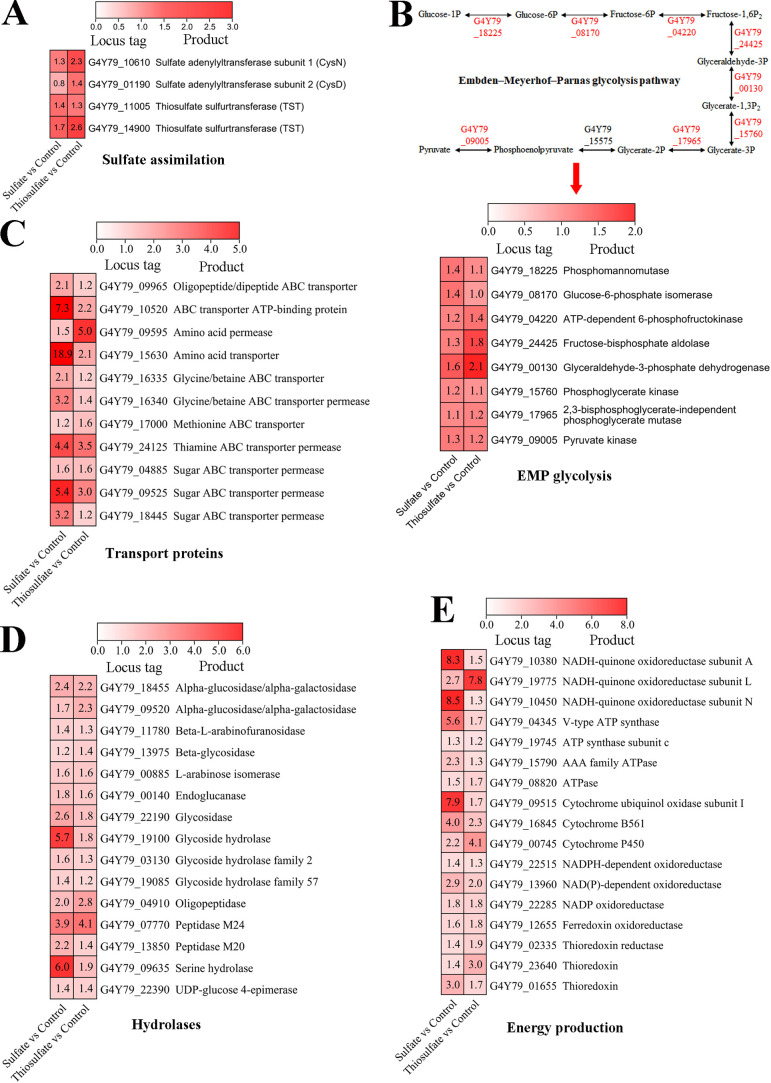
Proteomic analysis of *P. methaneseepsis* ZRK33 cultured in rich medium supplemented with sulfate and thiosulfate. (**A**) All upregulated proteins associated with sulfate assimilation. (**B**) Upregulated proteins associated with the EMP glycolysis pathway. (**C**) Upregulated proteins associated with amino acids and sugar transporters. (**D**) Upregulated proteins associated with saccharides/amino acids/peptides hydrolases. (**E**) Upregulated proteins associated with energy conversion. “Control” indicates the control group, where strain ZRK33 was cultured in rich medium alone; “Sulfate” indicates the sulfate-treated group, where strain ZRK33 was cultured in rich medium supplemented with 100 mM Na_2_SO_4_; and “Thiosulfate” indicates the thiosulfate-treated group, where strain ZRK33 was cultured in rich medium supplemented 100 mM Na_2_S_2_O_3_.

To better understand the sulfate assimilation of strain ZRK33, we performed proteomic analysis of strain ZRK33 cultured in rich medium alone or supplemented with either Na_2_SO_4_ or Na_2_S_2_O_3_ to explore the underlying mechanism of growth promotion. The expression of several proteins associated with sulfate assimilation, including sulfate adenylyltransferase subunit 1 (CysN), sulfate adenylyltransferase subunit 2 (CysD), and two thiosulfate sulfurtransferases (TST), was upregulated in the presence of Na_2_SO_4_ and Na_2_S_2_O_3_ (Fig. 3A; Table S2). In particular, both TSTs were upregulated in the presence of high concentrations of Na_2_SO_4_ and Na_2_S_2_O_3_, especially Na_2_S_2_O_3_, which was a key enzyme catalyzing S_2_O_3_^2−^ to SO_3_^2−^ and therefore important for sulfur assimilation (Fig. S1) (27). However, the expression of other proteins associated with assimilatory sulfate reduction was not detected, partly due to the single sampling time point which might be inappropriate to detect the upregulation of key proteins associated with sulfate assimilation. In addition, the expression of almost all genes involved in the Embden–Meyerhoff-Parnas (EMP) glycolysis pathway was upregulated (Fig. 3B), suggesting that strain ZRK33 might use the EMP glycolysis pathway to obtain energy for growth in the presence of Na_2_SO_4_ and Na_2_S_2_O_3_. Correspondingly, the expression of many proteins associated with organic matter metabolism to energy production was evidently upregulated, including amino acids and sugar ABC transporters (Fig. 3C), saccharides/peptides/amino acids degradation (Fig. 3D), and energy production (Fig. 3E). We therefore concluded that the metabolism of Na_2_SO_4_ and Na_2_S_2_O_3_ by strain ZRK33 could accelerate the hydrolysis and uptake of saccharides and other organic matter, thereby synthesizing energy to promote growth (28). Taking into account these results, we believe that strain ZRK33 possesses the capability to assimilate inorganic sulfur-containing compounds (e.g., sulfate and thiosulfate) that exist ubiquitously in deep-sea environments, thereby contributing to deep-sea sulfur cycling to some extent.

### *In situ* transcriptomic analysis of *P*. *methaneseepsis* ZRK33 cultured in deep-sea conditions

**Fig 4:**
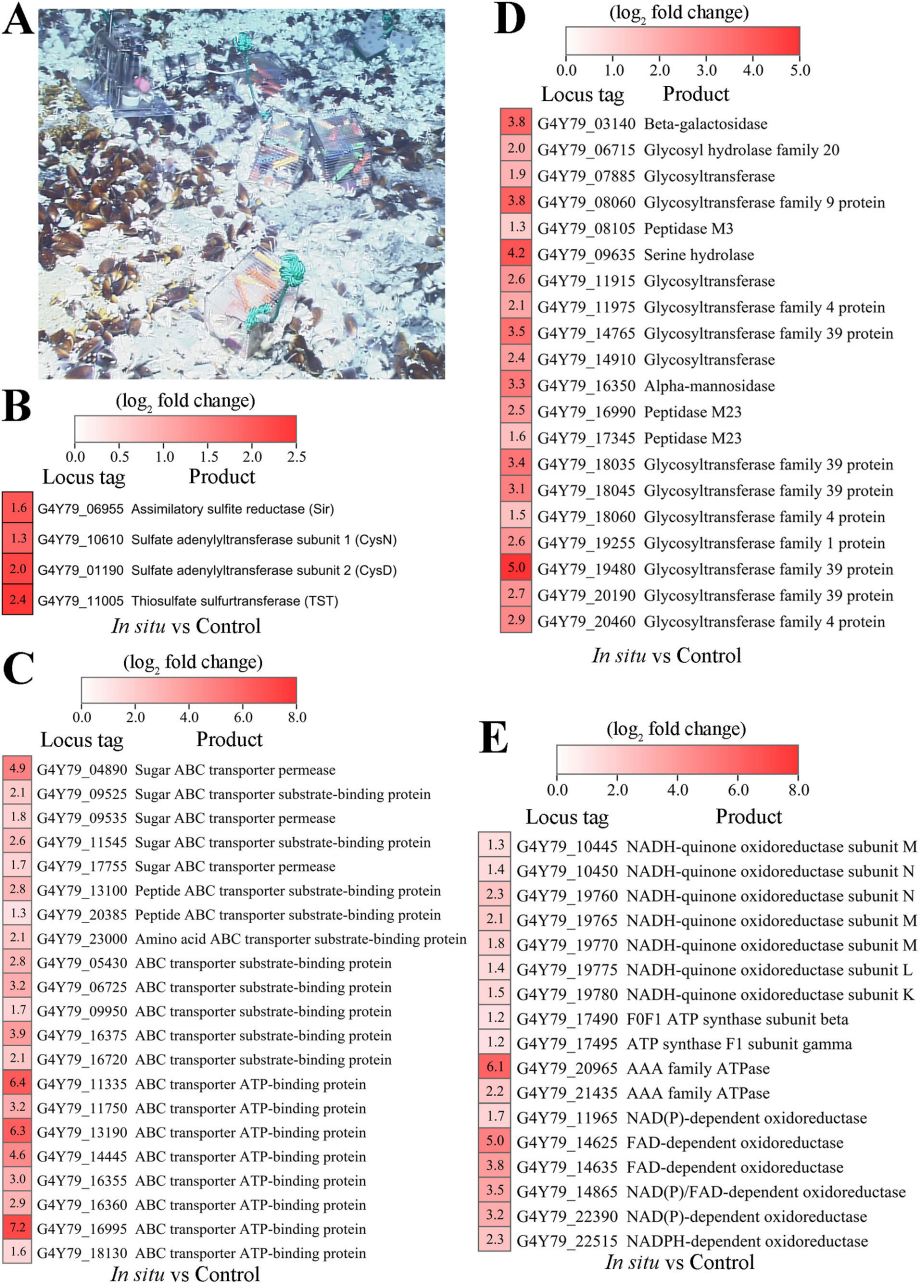
*In situ* transcriptomic analysis of *P. methaneseepsis* ZRK33 strains incubated in the deep-sea cold seep. (**A**) Close-up views of the *in situ* experimental apparatus in the deep-sea cold seep where many mussels and shrimps were distributed. (**B**) All upregulated genes encoding enzymes with sulfate assimilation. (**C**) Upregulated genes encoding proteins associated with sugar/amino acid/peptide ABC transporters. (**D**) Upregulated genes encoding enzymes with saccharides/amino acids/peptides hydrolases. (**E**) Upregulated genes encoding proteins associated with energy production. “Control” indicates strain ZRK33 cultivated in a deep-sea cold seep without exchange with outside; “*In situ*” indicates strain ZRK33 cultivated in a deep-sea cold seep with exchange with outside.

Considering strain ZRK33 was isolated from the deep-sea environment, we next sought to explore its metabolism when in the deep sea. We thus performed *in situ* cultivation of strain ZRK33 in the deep-sea cold seep (where we isolated this bacterium) for 10 days (Fig. 4A), as previously described (28). Subsequently, strain ZRK33 cells were collected and we performed a transcriptomic sequencing analysis. We found that the genes encoding sulfite reductase (Sir), sulfate adenylyltransferase subunit 1 (CysN), sulfate adenylyltransferase subunit 1 (CysD), and thiosulfate sulfurtransferase (TST) were upregulated (Fig. 4B; Table S3), consistent with laboratory conditions, indicating assimilatory sulfate reduction indeed occurred in the deep sea. Notably, genes encoding ABC transporters (associated with amino acids, sugars, and ions), glycoside hydrolases, and glycosyltransferases were upregulated (Fig. 4C and D), indicating that strain ZRK33 effectively ingests and degrades organic compounds with a coupled sulfate reduction process in the deep-sea environment (29, 30). In addition, genes encoding proteins associated with energy production (such as NADH-quinone oxidoreductase, ATP synthase, and FAD-dependent oxidoreductase) were also upregulated (Fig. 4E), also consistent with the laboratory conditions. The NADH-quinone oxidoreductase complex couples the oxidation of NADH and the reduction of quinone to generate a proton gradient, which is then used for ATP synthesis (31). In anoxic environments, sulfate-reducing bacteria are primarily responsible for organic carbon oxidation, because sulfate is often the predominant electron acceptor (32, 33). It has previously been reported that sulfate reduction can help facilitate organic matter oxidation up to 50% in marine sediments (34). Therefore, these results showed that deep-sea *Chloroflexota* bacteria might also contribute to the oxidation of organic matter in the deep sea sediments by assimilatory sulfate reduction, which could allow them to metabolically thrive in extreme habitats.

### A central metabolic model of *P*. *methaneseepsis* ZRK33

**Fig 5:**
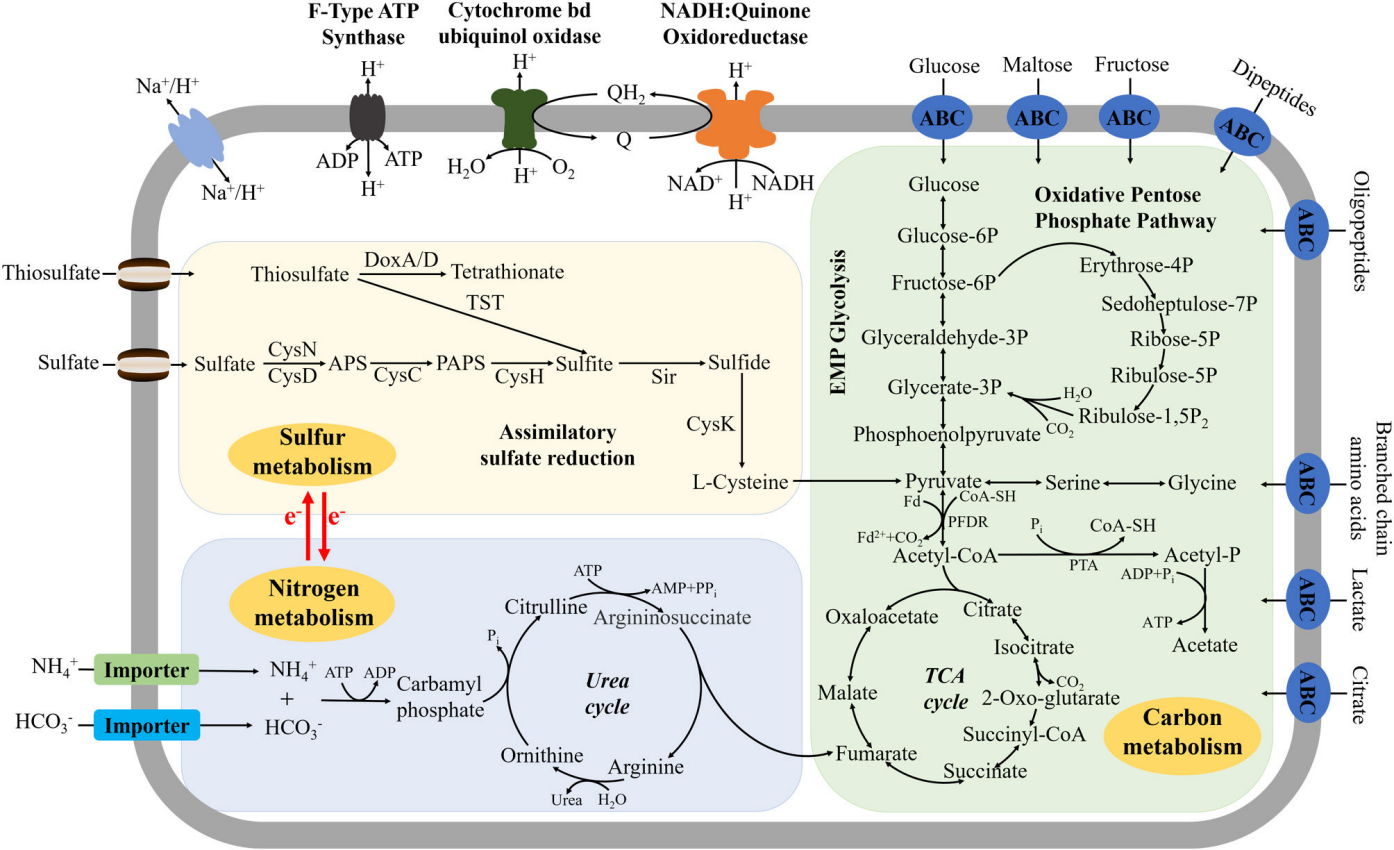
A muti-omics based central metabolism model of *P. methaneseepsis* ZRK33. In this model, three central metabolic pathways (associated with carbon metabolism, sulfur metabolism, and nitrogen metabolism) including EMP glycolysis, oxidative pentose phosphate pathway, TCA cycle, urea cycle, assimilatory sulfate reduction, and some electron transport systems are shown highlighted with different colors. All the above items are closely related to energy production in *P. methaneseepsis* ZRK33. TCA, tricarboxylic acid cycle; Urea, urea cycle; ATP, 5′-Adenylate triphosphate; ADP, adenosine diphosphate; AMP, adenosine monophosphate; Q, quinone; QH_2_, ubiquinone; CysN, sulfate adenylyltransferase subunit 1; CysD, sulfate adenylyltransferase subunit 2; CysC, adenylyl-sulfate kinase; CysH, phosphoadenosine phosphosulfate reductase; Sir, sulfite reductase; CysK, cysteine synthase; TST, thiosulfate sulfurtransferase; DoxA, thiosulfate dehydrogenase (quinone) small subunit; DoxD, thiosulfate dehydrogenase (quinone) large subunit.

Based on a combination of genomic, proteomic, transcriptomic, and physiological characteristics, we propose a model for the central metabolic traits of strain ZRK33 (Fig. 5). In this model, central metabolism is shown including the EMP glycolysis pathway, oxidative pentose phosphate pathway, tricarboxylic acid (TCA) cycle, assimilatory sulfate reduction, urea cycle, and electron transport system. All the above items are closely related to energy production in strain ZRK33. Briefly, strain ZRK33 contains a number of genes related to amino acid, peptide, and sugar ABC transporters, which could transport this organic matter into the cell to participate in the EMP glycolysis pathway and oxidative pentose phosphate pathway. These processes eventually drive the formation of pyruvate and acetyl-CoA, which enter into the TCA cycle to produce energy for the growth of strain ZRK33. As for the presence of all genes of the TCA cycle in the anaerobic strain ZRK33, we propose that it might use other alternative electron acceptors (such as sulfate reducers, nitrate reducers, and iron reducers) in place of oxygen for the TCA cycle, as shown in other anaerobic bacteria (35). Of note, sulfate and thiosulfate could be converted to cysteine and thereby enter the pyruvate synthesis pathway through assimilatory sulfate reduction, which might promote saccharide degradation and utilization via unknown mechanisms. Moreover, strain ZRK33 could transport ammonium and bicarbonate ions into the cell to be catalyzed into carbamyl phosphate into the urea cycle, and corresponding metabolites could join the TCA cycle for energy generation. Meanwhile, also present in the genome of strain ZRK33 are the F-type ATP synthase, cytochrome *bd* ubiquinol oxidase and H^+^-transporting NADH: Quinone oxidoreductase required for energy production. In addition, strain ZRK33 could not fix CO_2_ and perform chemoautotrophic or photoautotrophic growth, with growth only possible in the presence of organic carbon compounds. *In situ* transcriptomic results showed that many genes encoding for phototrophy-relevant enzymes (our previous study) (22) and carbon/sulfur metabolism enzymes (Fig. 4) are expressed concurrently, suggesting that strain ZRK33 could perform a mixotrophic lifestyle to cope with the extreme deep-sea environment. Accordingly, we found that red light [wavelengths of 620–625 nm (80 µmol m^−2^ s^−1^)] and infrared light [wavelengths of 940 nm (5 µmol m^−2^ s^−1^)] (36) could evidently promote the growth of strain ZRK33 (Fig. S3). Actually, there is some evidence showing that both long wavelength (>650 nm) (37) and short wavelength (<650 nm) light have been detected in deep sea (38). With that, we infer that the light existing in the deep-sea environments should promote the growth of strain ZRK33 to some extent, as described in our previous report (22). Considering there is no abundant organic nutrition in the deep sea, we propose that strain ZRK33 does not use light-derived energy to fix CO_2_ but to supplement its heterotrophic metabolism. Overall, strain ZRK33 is a representative of the phylum *Chloroflexota* possessing diverse metabolic pathways for energy production, providing evidence that *Chloroflexota* members are high-abundance bacteria ubiquitously distributed in different environments.

### Metagenomic and metatranscriptomic analysis of sulfur metabolism in deep-sea *Chloroflexota* bacteria

**Fig 6:**
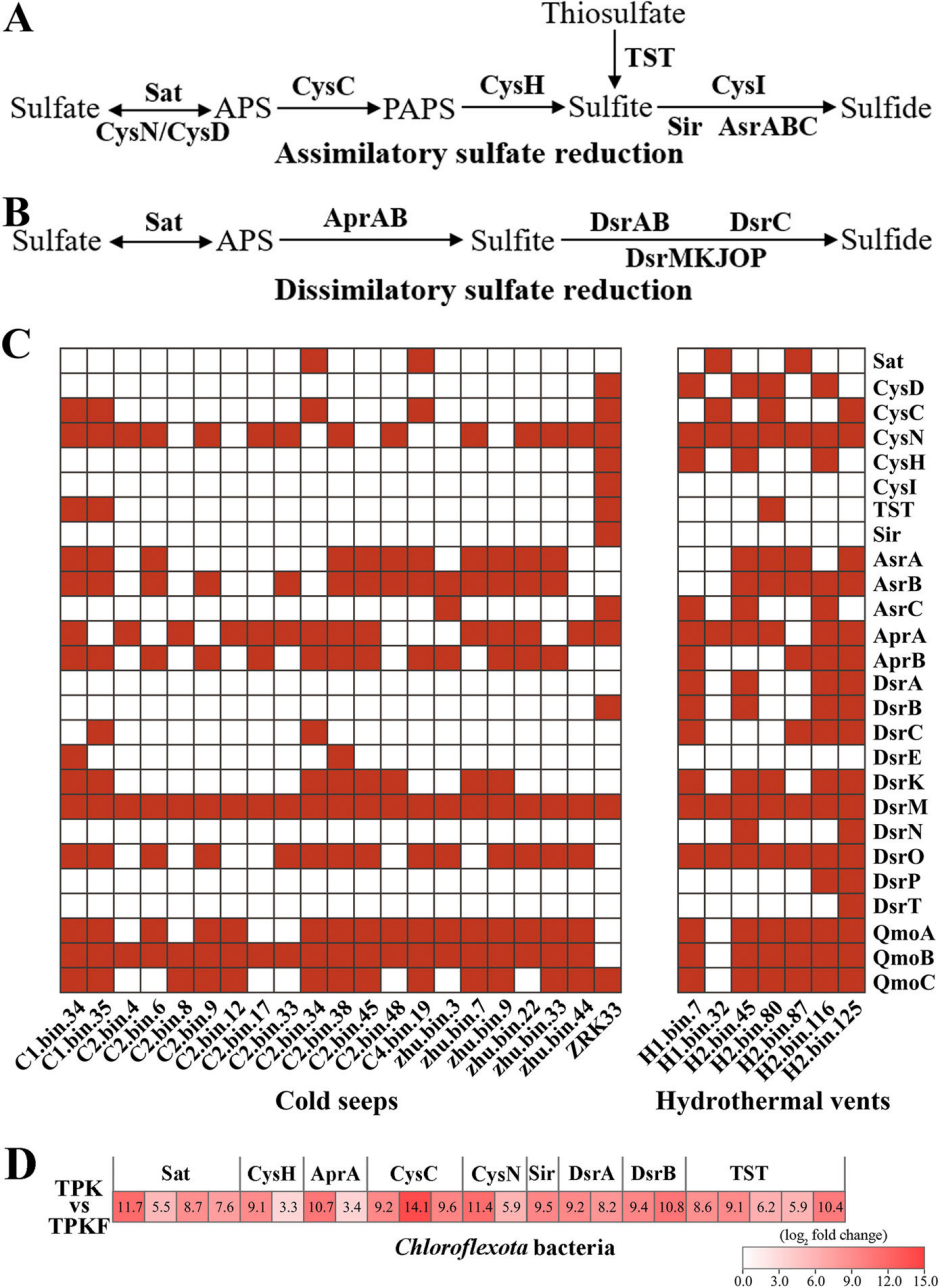
Metagenomic and metatranscriptomic analysis of sulfur metabolism in deep-sea *Chloroflexota* bacteria. (**A**) Typical pathway of assimilatory sulfate reduction existing in bacteria. (**B**) Typical pathway of dissimilatory sulfate reduction existing in bacteria. (**C**) Distribution of genes encoding key enzymes involved in assimilatory and dissimilatory sulfate reduction in deep-sea *Chloroflexota* MAGs and strain ZRK33. The presence of enzymes involved in the sulfur metabolic pathway is indicated by using red-colored rectangles. Sat, sulfate adenylyltransferase; CysN, sulfate adenylyltransferase subunit 1; CysD, sulfate adenylyltransferase subunit 2; CysC, adenylyl-sulfate kinase; CysH, phosphoadenosine phosphosulfate reductase; Sir, sulfite reductase; AsrA, AsrB, and AsrC, anaerobic sulfite reductases; CysI, sulfite reductase (NADPH) hemoprotein beta-component; TST, thiosulfate sulfurtransferase; AprA and AprB, adenylylsulfate reductase; DsrA and DsrB, dissimilatory sulfite reductase; DsrC, protein substrate of dissimilatory sulfite reductase; DsrMKJOP, sulfite reduction-associated complex. (**D**) *In situ* metatranscriptomics-based heat map showing upregulated genes encoding proteins associated with sulfur metabolism in deep-sea *Chloroflexota* bacteria. The numbers in panel (D) represent the fold change of gene expression (using log_2_ values). TPK, sediment from the center of cold seep; TPKF, sediment far away from the center of cold seep.

To evaluate the contribution of *Chloroflexota* bacteria to deep-sea sulfur cycling, we further analyzed the distribution of genes encoding key enzymes responsible for both assimilatory sulfate reduction (Fig. 6A) and dissimilatory sulfate reduction (Fig. 6B) in 27 metagenome-assembled genomes (MAGs) of *Chloroflexota* bacteria derived from both deep-sea cold seep and hydrothermal vent sediments. Assembly statistics and quality metrics of the reconstructed genome bins of *Chloroflexota* are shown in Table S4. We found that diverse genes encoding key enzymes in charge of assimilatory and dissimilatory sulfate reduction (including CysC, CysN, AsrABC, DsrAB, DsrC, the DsrMK complex, and the QmoABC complex) were widely distributed in both cold seep and hydrothermal vent derived MAGs (Fig. 6C). Of note, genes encoding Sat, DsrAB, DsrC, the DsrMK complex, and the QmoABC complex were broadly present in the hydrothermal vent-derived MAGs (Fig. 6C). DsrA and DsrB are typical symbols of microbes mediating dissimilatory sulfate reduction (4). DsrAB could produce a DsrC-trisulfide from the sulfite and DsrC protein. Then, the DsrC-trisulfide is reduced by the DsrMK(JOP) membrane complex, which recycles DsrC and releases sulfide while coupling this reduction to energy conservation (39–41). Thus, the DsrABCMK is defined as a minimal set of proteins necessary for dissimilatory sulfite reduction (42). In addition, the QmoABC complex (for Quinone-interacting membrane-bound oxidoreductase) was first described in *D. desulfuricans* ATCC 27774 (43), and was necessary for dissimilatory sulfate reduction, but not for sulfite reduction (44). Therefore, we propose that dissimilatory sulfate reduction might often be adopted by members of *Chloroflexota* in hydrothermal vents, which differs from recent research on *Chloroflexota* from hydrothermal vents where some members had a capacity for sulfide oxidation (45), suggesting that *Chloroflexota* in different hydrothermal vents could engage in different sulfur metabolism processes. In combination with reports that the other two *Chloroflexota* lineages (SAR202 group and *Dehalococcoidia* class) possess the potential to drive sulfur metabolism (4, 21), it is reasonable to suggest that the phylum *Chloroflexota* greatly contributes to ocean sulfur cycling.

Given the high abundance of *Chloroflexota* bacteria and the true sulfate assimilation of strain ZRK33 in the deep-sea cold seep, we performed metatranscriptomic sequencing to investigate the metabolism of other deep-sea *Chloroflexota* bacteria. This showed that genes encoding multiple enzymes associated with assimilatory sulfate reduction and dissimilatory sulfate reduction (such as Sat, CysH, AprA, CysC, CysN, Sir, DsrA, DsrB, and TST) were upregulated in the center of the cold seep sediment (where strain ZRK33 was isolated) (Fig. 6D). This indicates that *Chloroflexota* bacteria indeed perform sulfate reduction in the deep-sea environment and play a pivotal role in sulfur biogeochemical cycling.

In summary, our findings indicate that the deep-sea *Chloroflexota* bacterium ZRK33 can perform assimilatory sulfate reduction in both laboratory and deep-sea conditions, which expands our knowledge of the sulfur metabolic potential of deep-sea *Chloroflexota* bacteria and provides hints to roles for *Chloroflexota* bacteria in the marine sedimentary sulfur cycle. Important information is also provided regarding the broad distribution and the upregulated expression of diverse genes related to sulfur metabolism in deep-sea *Chloroflexota* bacteria, suggesting that *Chloroflexota* bacteria play a pivotal role in the deep-sea sulfur biogeochemical cycling.

## MATERIALS AND METHODS

### Growth assays of strain ZRK33

To assess the effect of yeast extract and peptone on the growth of strain ZRK33, 30 mL of freshly incubated cells were inoculated in 1.5 L of either basal medium [containing 1.0 g/L NH_4_Cl, 1.0 g/L NaHCO_3_, 1.0 g/L CH_3_COONa, 0.5 g/L KH_2_PO_4_, 0.2 g/L MgSO_4_.7H_2_O, 0.7 g/L cysteine hydrochloride, and 500 µL/L 0.1% (wt/vol) resazurin, pH 7.0] alone, basal medium supplemented with 0.5 g/L, 1.0 g/L, 5.0 g/L, or 10.0 g/L yeast extract and peptone at 28°C for 12 days. To assess the effects of different inorganic sulfur sources (20 mM Na_2_SO_4_, 100 mM Na_2_SO_4_, 20 mM Na_2_S_2_O_3_, 100 mM Na_2_S_2_O_3_, 1 mM Na_2_SO_3_, and 1 mM Na_2_S) on strain ZRK33 growth, we used a rich medium [containing 1.0 g/L yeast extract, 1.0 g/L peptone, 1.0 g/L NH_4_Cl, 1.0 g/L NaHCO_3_, 1.0 g/L CH_3_COONa, 0.5 g/L KH_2_PO_4_, 0.2 g/L MgSO_4_.7H_2_O, 0.7 g/L cysteine hydrochloride, and 500 µL/L 0.1% (wt/vol) resazurin, pH 7.0] supplemented with the sulfur sources mentioned above. To assess the effects of red light and infrared light on the growth of strain ZRK33, we cultured it in the conditions exposed red light [wavelengths of 620–625 nm (80 µmol m^−2^ s^−1^)], infrared light [wavelengths of 940 nm (5 µmol m^−2^ s^−1^)], and darkness. For each growth assay, 30 mL of strain ZRK33 culture was inoculated in a 2-L Hungate bottle containing 1.5 L of the respective media. All Hungate bottles were anaerobically incubated at 28°C for 12 days. Bacterial growth was monitored by measuring daily OD_600_ values via a microplate reader until cell growth reached a stationary phase. Three replicates were performed for each condition. For the determination of the dynamics of the concentrations of Na_2_SO_4_ and Na_2_S_2_O_3_ in the culture, we selected three cultivation time points at 5, 8, and 12 days, respectively, and each condition had three replicates. The supernatant was collected at 12,000 *× g* for 10 min and diluted 80 times, and the concentrations of SO_4_^2−^ and S_2_O_3_^2−^ in the diluted supernatant were respectively measured by the ion chromatograph (ECO IC, Herisau, Switzerland) with a chromatographic column (Metrosep A Supp5). The column was eluted with mobile phase A (3.2 mmol/L Na_2_CO_3_) and mobile phase B (1.0 mmol/L NaHCO_3_) at 25°C.

### Scanning electron microscope observation

To observe the morphological characteristics of strain ZRK33, 10 µL of sample (ZRK33 cells) was dripped on coverslips and soaked in gelatin and dried for 30 min to allow the sample to adhere to the surface of copper grid. These samples were fixed in 2.5% glutaraldehyde for 30 min. Samples were then washed three times with phosphate-buffered saline (PBS) and dehydrated in ethanol solutions of 30%, 50%, 70%, 90%, and 100% for 10 min each time. All samples were observed with SEM (S-3400N, Hitachi, Japan) at 5 kV.

### Transmission electron microscope observation

To observe the morphological characteristics of strain ZRK33, the cell suspension of fresh culture was collected at 5,000 × *g* for 10 min and washed with Milli-Q water. The cells were then collected by immersing copper grids coated with a carbon ﬁlm in the cell suspension for 20 min. The copper grids were then washed for 10 min in Milli-Q water and dried for 20 min at room temperature. Finally, the sample was examined using TEM (HT7700, Hitachi, Japan) with a JEOL JEM 1200 EX (equipped with a ﬁeld emission gun) at 100 kV.

### Proteomic analysis

Proteomic sequencing analysis was performed by PTMBiolabs (Hangzhou, China). Briefly, strain ZRK33 was respectively cultivated in the rich medium (set as the control group and indicated as “Control”), rich medium supplemented with 100 mM Na_2_SO_4_ (set as the experimental group and indicated as “Sulfate”) and 100 mM Na_2_S_2_O_3_ (set as the experimental group and indicated as “Thiosulfate”) at 28°C for 8 days. Then, the cells were collected and sonicated three times on ice using a high-intensity ultrasonic processor in lysis buffer (8 M urea, 1% Protease Inhibitor Cocktail). The remaining debris was removed by centrifugation at 12,000 × *g* at 4°C for 10 min. Finally, the supernatant was collected and the protein concentration was determined with a BCA kit (Solarbio, China) according to the instructions. The detailed protocols of proteomics sequencing technology were described in the Supplementary information.

### Transcriptomic analysis of the ZRK33 strain incubated in a deep-sea cold seep

To explore the metabolism of strain ZRK33 in the deep-sea cold seep, the strain was initially cultured in a rich medium for 7 days. Following this, 30 mL of the fresh cultures was then transferred to 1.5 L of the rich medium. Thereafter, the 1.5 L culture medium of strain ZRK33 was divided into two parts: one part was divided and transferred equally into three gas sample bags (which not allowing any exchanges between inside and outside; aluminum-plastic composite film, Hede, China) with 200 mL culture medium each and set as control groups; the other part was divided into three dialysis bags (8,000–14,000 Da cutoff, which allowing the exchanges of substances smaller than 8,000 Da but preventing bacterial cells from entering or leaving the bag; Solarbio, China) with 200 mL culture medium each and set as experimental groups. All samples were placed simultaneously in the deep-sea cold seep, where strain ZRK33 was isolated for 10 days in June 2020 during the cruise of *Kexue* vessel. After 10 days of *in situ* incubation, the bags were recycled and the cells were immediately collected and kept at −80°C for future analysis. The cells were checked by 16S rRNA sequencing to confirm the purity of the cultures and subsequently investigated further by transcriptomics analysis. The detailed protocols for transcriptomic sequencing analysis were conducted as previously described (46).

### Metagenomic sequencing, assembly, binning, and annotation

To understand the sulfur metabolic characteristics of deep-sea *Chloroflexota* bacteria, four cold seep sediment samples (zhu, C1, C2, and C4) and two hydrothermal vents sediment samples (H1 and H2) were selected for metagenomic analysis in BGI (BGI, China). Briefly, total DNAs from these samples (20 g each) were extracted using the Qiagen DNeasy PowerSoil Pro Kit (Qiagen, Hilden, Germany) and the integrity of DNA was evaluated by gel electrophoresis. Then, 0.5 µg DNA of each sample was used for library construction. The library was prepared with an amplification step for each sample. And then DNAs were cleaved into 50–800 bp fragments by the Covaris E220 ultrasonicator (Covaris, Brighton, UK) and some fragments between 150 and 250 bp were selected using AMPure XP beads (Agencourt, USA) and repaired using T4 DNA polymerase (Enzymatics, USA). All next-generation sequencing (NGS) was performed on the BGISEQ-500 platform (BGI, Qingdao, China) and generated 100 bp paired-end raw reads. Quality control was performed by SOAPnuke (v1.5.6) (setting: -l 20 -q 0.2n 0.05 -Q 2 -d -c 0–5 0–7 1) (47) and the clean data were assembled using MEGAHIT (v1.1.3) (setting:--min-count 2k-min 33 --k-max 83 --k-step 10) (48). Assemblies of these samples were automatically binned using Maxbin2 (version 2.2.7, -markerset 40 option) (49), metaBAT2 (version 2.12.1, -m 1500 and --unbinned parameters) (50) and Concoct (version 0.4.0, default settings) (51). MetaWRAP (version 1.3.2, default parameters) (52) was used to purify and organize data to generate the final bins. Finally, the completeness and contamination of MAGs were assessed by the checkM (v1.0.18) (53). These obtained MAGs were subsequently annotated by searching these predicted genes against KEGG (Release 87.0), NR (20180814), Swissprot (release-2017_07), and COG (update-2018_08) databases. Additionally, to search for genes associated with sulfur metabolism in these MAGs and strain ZRK33, the hmmsearch (HMMER 3.0 with the default parameters and e-value cut-off of 1e-20) was used. When performing HMM searches, the Pfam (version 33.1) (54) and TIGRFAM (Release 15.0, http://www.jcvi.org/tigrfams) (55, 56) databases were used to search for target genes. Some enzymes for interest pathways were further searched by running tBLASTn against the relevant reference sequences from NCBI database. A tBLASTn hit with the sequence identity ≥30%, an *e*-value ≤ e−6, and a percent alignment length ≥30% was considered a potential homolog (57).

### Metatranscriptomic analysis

To explore the actual metabolic characteristics of deep-sea *Chloroflexota* bacteria conducted in the deep-sea cold seep, the *in situ* metatranscriptomic analysis was performed. Two cold seep sediment samples (TPK, sediment from the center of cold seep; TPKF, sediment far away from the center of cold seep) were selected for metatranscriptomic sequencing analysis in Shanghai Biozeron Biotechnology Co., Ltd. (Shanghai, China). Total RNAs were extracted from these sediments using TRIzol Reagent according to the manufacturer’s instructions and genomic DNA was removed using DNase I (TaKara). Then, RNA quality was determined using 2100 Bioanalyzer (Agilent) and quantified using the ND-2000 (NanoDrop Technologies). A high-quality RNA sample (OD260/280 = 1.8–2.2, OD260/230 ≥ 2.0, RIN ≥ 6.5, 28S:18S ≥ 1.0, >10 µg) is used to construct the sequencing library. The detailed protocols of library preparation, Illumina Hiseq sequencing, reads quality control and mapping, metatranscriptome assembly and annotation, and data analyses are described in the Supplementary information.

## Data Availability

The BioProject accession number of metagenome-assembled genomes (MAGs) of *Chloroflexota* bacteria used in this study is PRJNA667788. The complete genome sequence of strain ZRK33 has been deposited at GenBank under the accession number CP062983. The mass spectrometry proteomics data have been deposited to the Proteome Xchange Consortium with the data set identifier PXD023380. The raw sequencing reads from the transcriptomics analysis have been deposited to the NCBI Short Read Archive (accession number: PRJNA1012367). The raw metatranscriptomic sequencing data have been deposited to NCBI Short Read Archive (accession number: PRJNA988008).

## SUPPLEMENTAL MATERIAL

Fig. S1 mbio.00004-24-s0001.docx Proposed pathway of assimilatory sulfate reduction existing in *P. methaneseepsis* ZRK33.

Fig. S2 mbio.00004-24-s0002.docx Growth assays of *P. methaneseepsis* ZRK33 cultured in rich medium supplemented with different sulfur-containing compounds.

Fig. S3 mbio.00004-24-s0003.docx Growth assay of *P. methaneseepsis* ZRK33 cultivated in the rich medium exposed to darkness and different wavelengths of light illumination.

Supplemental methods mbio.00004-24-s0004.docx Detailed procedures of proteomic and metatranscriptomic analyses.

Table S1 mbio.00004-24-s0005.docx The genomic analysis of *P. methaneseepsis* ZRK33.

Table S2 mbio.00004-24-s0006.docx The proteomic analysis of *P. methaneseepsis* ZRK33 cultured in rich medium alone or supplemented with either Na_2_SO_4_ or Na_2_S_2_O_3_.

Table S3 mbio.00004-24-s0007.docx *In situ* transcriptomic analysis of *P. methaneseepsis* ZRK33 cultured in deep-sea conditions.

Table S4 mbio.00004-24-s0008.docx Assembly statistics and quality metrics of reconstructed genome bins of Chloroflexota used in this study.

ASM does not own the copyrights to Supplemental Material that may be linked to, or accessed through, an article. The authors have granted ASM a non-exclusive, world-wide license to publish the Supplemental Material files. Please contact the corresponding author directly for reuse.
