# Supplementary material for: Deep-sea in situ and laboratory multi-omics provide insights into the sulfur assimilation of a deep-sea Chloroflexota bacterium
Source: mBio. 2024 Feb 28;15(4):e00004-24. doi: 10.1128/mbio.00004-24 (PMC11005417; doi:10.1128/mbio.00004-24)
Supplement: Fig. S1 — Proposed pathway of assimilatory sulfate reduction existing in P. methaneseepsis ZRK33. [file mbio.00004-24-s0001.docx]

**
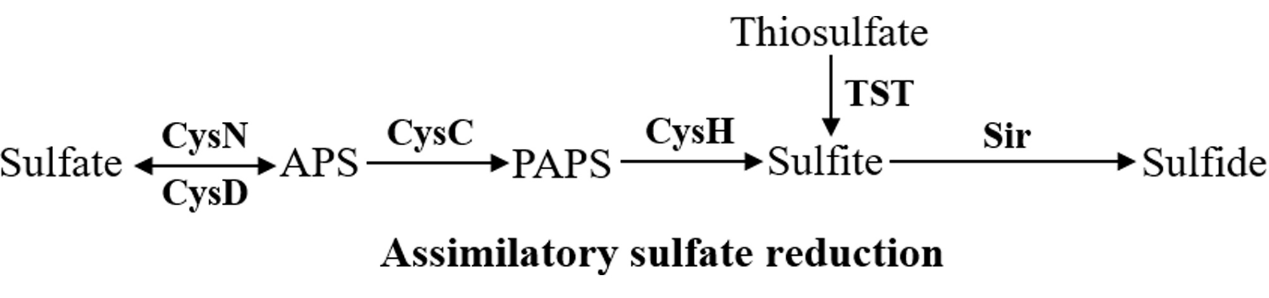
**

**Supplementary FIG S1. Proposed pathway of assimilatory sulfate reduction existing in *P*. *methaneseepsis* ZRK33.** Abbreviations: CysN, sulfate adenylyltransferase subunit 1; CysD, sulfate adenylyltransferase subunit 2; CysC, adenylyl-sulfate kinase; CysH, phosphoadenosine phosphosulfate reductase; Sir, sulfite reductase; TST, thiosulfate sulfurtransferase.
