# Supplementary material for: Deep-sea in situ and laboratory multi-omics provide insights into the sulfur assimilation of a deep-sea Chloroflexota bacterium
Source: mBio. 2024 Feb 28;15(4):e00004-24. doi: 10.1128/mbio.00004-24 (PMC11005417; doi:10.1128/mbio.00004-24)
Supplement: Fig. S2 — Growth assays of P. methaneseepsis ZRK33 cultured in rich medium supplemented with different sulfur-containing compounds. [file mbio.00004-24-s0002.docx]

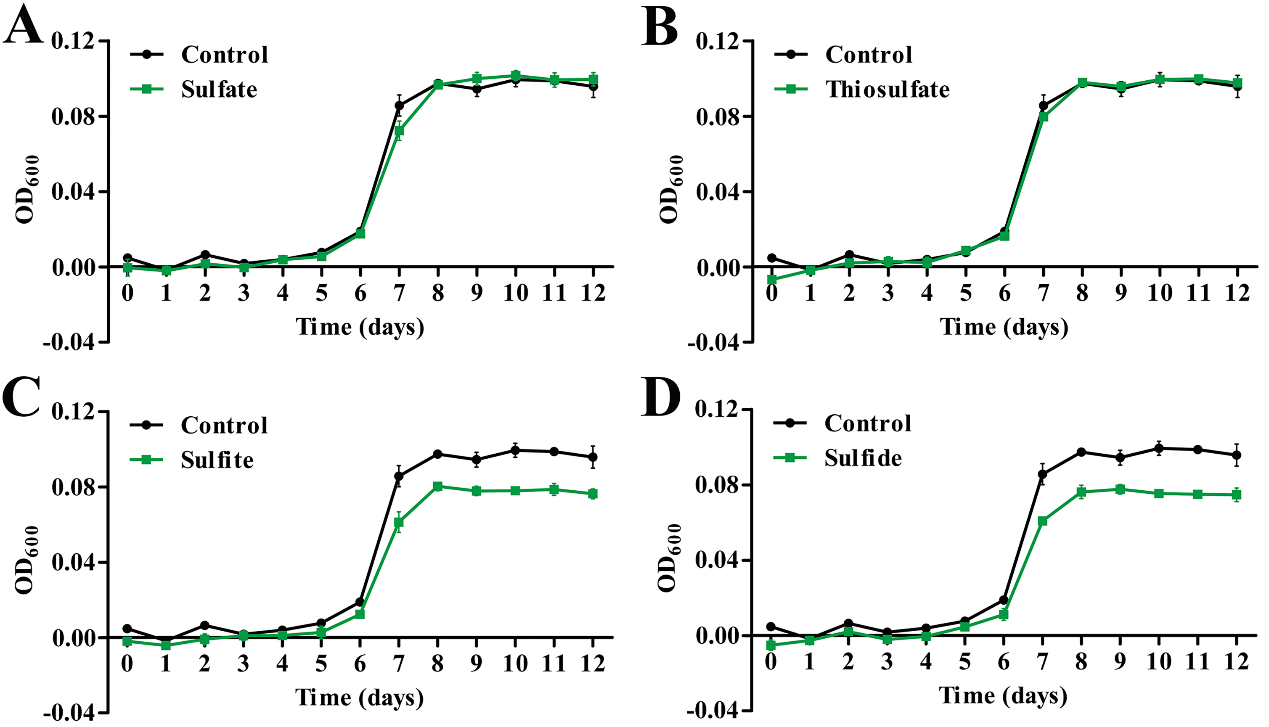


**Supplementary FIG S2. Growth assays of *P*. *methaneseepsis* ZRK33 cultured in rich medium supplemented with different sulfur-containing compounds.** (A) Growth assays of strain ZRK33 in the medium supplemented without or with 20 mM Na_2_SO_4_. (B) Growth assays of strain ZRK33 in the medium supplemented without or with 20 mM Na_2_S_2_O_3_. (C) Growth assays of strain ZRK33 in the medium supplemented without or with 1 mM Na_2_SO_3_. (D) Growth assays of strain ZRK33 in the medium supplemented without or with 1 mM Na_2_S. “Control” indicates the control group, where strain ZRK33 was cultured in rich medium alone; “Sulfate” indicates the sulfate-treated group, where strain ZRK33 was cultured in rich medium supplemented with 20 mM Na_2_SO_4_; “Thiosulfate” indicates the thiosulfate-treated group, where strain ZRK33 was cultured in rich medium supplemented with 20 mM Na_2_S_2_O_3_; “Sulfite” indicates the sulfite-treated group, where strain ZRK33 was cultured in rich medium supplemented with 1 mM Na_2_SO_3_; “Sulfide” indicates the sulfide-treated group, where strain ZRK33 was cultured in rich medium supplemented with 1 mM Na_2_S.
