# Supplementary material for: Deep-sea in situ and laboratory multi-omics provide insights into the sulfur assimilation of a deep-sea Chloroflexota bacterium
Source: mBio. 2024 Feb 28;15(4):e00004-24. doi: 10.1128/mbio.00004-24 (PMC11005417; doi:10.1128/mbio.00004-24)
Supplement: Fig. S3 — Growth assay of P. methaneseepsis ZRK33 cultivated in the rich medium exposed to darkness and different wavelengths of light illumination. [file mbio.00004-24-s0003.docx]

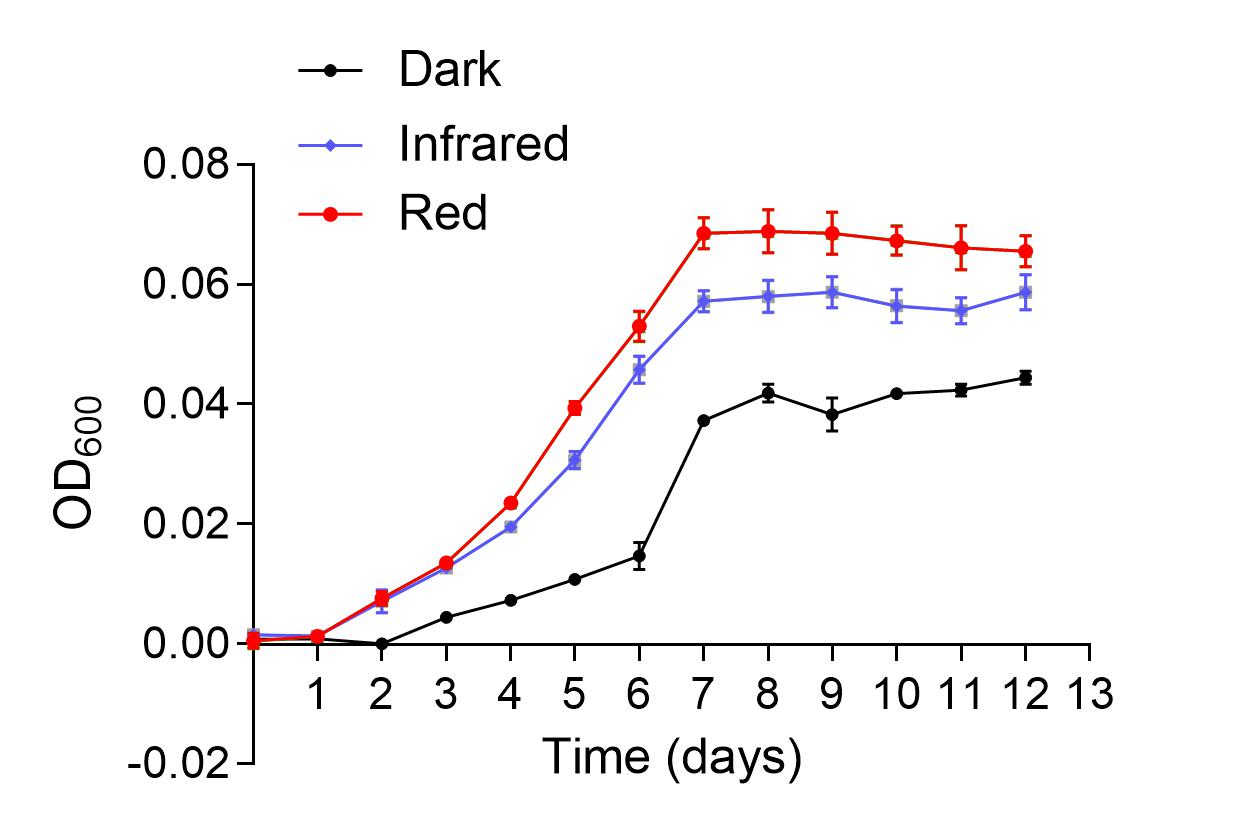


**Supplementary FIG S3. Growth assay of *P. methaneseepsis* ZRK33 cultivated in the rich medium exposed to darkness and different wavelengths of light illumination.** In this panel, **“**Dark” indicates darkness; **“**Red” indicates red light (wavelengths of 620-625 nm [80 μmol m^-2^ s^-1^]); “Infrared” indicates infrared light (wavelengths of 940 nm [5 μmol m^-2^ s^-1^]).
