# Supplementary material for: Deep-sea in situ and laboratory multi-omics provide insights into the sulfur assimilation of a deep-sea Chloroflexota bacterium
Source: mBio. 2024 Feb 28;15(4):e00004-24. doi: 10.1128/mbio.00004-24 (PMC11005417; doi:10.1128/mbio.00004-24)
Supplement: Supplemental methods — Detailed procedures of proteomic and metatranscriptomic analyses. [file mbio.00004-24-s0004.docx]

**Supplementary information**

**Deep-sea *in situ* and laboratory multi-omics provide insights into the sulfur assimilation of a deep-sea *Chloroflexota* bacterium**

Rikuan Zheng^1,2,3#^, Chong Wang^1,2,3#^, Chaomin Sun^1,2,3,4*^

^1^CAS and Shandong Province Key Laboratory of Experimental Marine Biology & Center of Deep Sea Research, Institute of Oceanology, Chinese Academy of Sciences, Qingdao, China

^2^Laboratory for Marine Biology and Biotechnology, Qingdao Marine Science and Technology Center, Qingdao 266071, China

^3^Center of Ocean Mega-Science, Chinese Academy of Sciences, Qingdao, China

^4^College of Earth Science, University of Chinese Academy of Sciences, Beijing 100049, China

^#^ Co-author

^*^ Corresponding author

Chaomin Sun Tel.: +86 532 82898857; fax: +86 532 82898857.

E-mail address: sunchaomin@qdio.ac.cn

**SUPPLEMENTAL MATERIAL**

**A detailed procedure for proteomic analysis of strain ZRK33**

**Sample processing protocol.** Strain ZRK33 was cultivated in the rich medium supplemented without or with 100 mM Na_2_SO_4_ or 200 mM Na_2_S_2_O_3_ for 8 d at 28 °C and then the cells were collected. These cells were sonicated three times on ice using a high intensity ultrasonic processor in lysis buffer (8 M urea, 1% Protease Inhibitor Cocktail). The remaining debris was removed by centrifugation at 12,000 × *g* at 4 °C for 10 min. Finally, the supernatant was collected and the protein concentration was determined with a BCA kit (Solarbio, China) according to the manufacturer's instructions. For trypsin digestion, the protein solution was reduced with 5 mM dithiothreitol for 30 min at 56 °C and alkylated with 11 mM iodoacetamide for 15 min at room temperature in darkness. The 100 mm TEAB was added to the diluted protein sample in a solution with a urea concentration of less than 2 M. Finally, trypsin was added at a trypsin to protein mass ratio of 1:50 for the first digestion overnight, with 1:100 trypsin and protein. The mass was added for a second digestion for 4 h. Then the tryptic peptides were dissolved in 0.1% formic acid (solvent A) and directly loaded into a home-made reversed-phase analytical column (15-cm length, 75 μm inner diameter). The gradient increased from 6% to 23% in solvent B (0.1% formic acid in 98% acetonitrile) over 26 min, from 23% to 35% in 8 min and increased to 80% in 3 min, then maintain 80% for the last 3 min, and all at a constant flow rate of 400 nL/min on an EASY-nLC 1000 UPLC system.

The peptides were coupled to UPLC in Q ExactiveTM Plus (Thermo, USA) via NSI source and tandem mass spectrometry (MS/MS). The applied electrospray voltage was 2.0 kV. The full scan has an m/z scan range of 350 to 1,800, and at 70,000 resolution, intact peptides were detected in the Orbitrap. MS/MS was then selected using the NCE set to 28 select peptides and fragments were detected in the Orbitrap at a resolution of 17,500. A data-related process that alternates between one MS scan followed by 20 MS/MS scans with 15.0 s dynamic exclusion. The automatic gain control (AGC) was set to 5E4. The fixed first mass was set as 100 m/z.

**Data processing protocol. (1) Database Search.** The resulting MS/MS data were processed using Maxquant search engine (v.1.5.2.8) (1). Tandem mass spectra were searched against the NCBI database concatenated with the reverse decoy database. Trypsin/P was specified as cleavage enzyme allowing up to 2 missing cleavages. The mass tolerance for precursor ions was set as 20 ppm in First search and 5 ppm in Main search, and the mass tolerance for fragment ions was set as 0.02 Da. Carbamidomethyl on Cys was specified as fixed modification and oxidation on Met was specified as variable modifications. FDR was adjusted to < 1% and minimum score for peptides was set > 40. The peptides sequences were annotated using KEGG (Release 97.0), Pfam (version 33.1), COG (version 2020), and Uniprot ([release 2022_01](https://www.uniprot.org/release-notes/2022-02-23-release)) (2-4). The cut‐off was set as 1e‐20. **(2) Enrichment of Gene Ontology analysis.** Proteins were classified by GO annotation into three categories: biological process, cellular compartment and molecular function. For each category, a two-tailed Fisher’s exact test was employed to test the enrichment of the differentially expressed protein against all identified proteins. The cut-off was set as 1e-20. The GO with a corrected *P*-value < 0.05 is considered significant. **(3) Enrichment of pathway analysis.** Encyclopedia of Genes and Genomes (KEGG) database (Release 97.0, https://www.genome.jp/kegg/) was used to identify enriched pathways by a two-tailed Fisher’s exact test to test the enrichment of the differentially expressed protein against all identified proteins (5). The cut-off was set as 1e-20. The pathway with a corrected *P*-value < 0.05 was considered significant. These pathways were classified into hierarchical categories according to the KEGG website. **(4) Enrichment of protein domain analysis.** For each category proteins, InterPro (a resource that provides functional analysis of protein sequences by classifying them into families and predicting the presence of domains and important sites) database (InterPro 87.0, https://www.ebi.ac.uk/interpro/) was researched and a two-tailed Fisher’s exact test was employed to test the enrichment of the differentially expressed protein against all identified proteins. The cut-off was set as 1e-20. Protein domains with a *P*-value < 0.05 were considered significant. **(5) Enrichment-based Clustering.** For further hierarchical clustering based on different protein functional classification (such as: GO, Domain, Pathway, Complex). We first collated all the categories obtained after enrichment along with their *P* values, and then filtered for those categories which were at least enriched in one of the clusters with *P* value <0.05. This filtered *P* value matrix was transformed by the function x = −log_10_ (*P* value). Finally these x values were z-transformed for each functional category. These z scores were then clustered by one-way hierarchical clustering (Euclidean distance, average linkage clustering) in Genesis. Cluster membership was visualized by a heat map using the “heatmap.2” function from the “gplots” R-package.

**The detailed procedure for metatranscriptomic analysis**

**Library preparation, and Illumina Hiseq sequencing.** Metatranscriptome libraries were prepared following TruSeq TM Stranded Total RNA Sample Preparation Kit from Illumina (San Diego, CA), using 5 μg of total RNA. Briefly, rRNA removal by Ribo-Zero TM rRNA Removal Kits from Illumina (San Diego, CA), fragmented using fragmentation buffer. cDNA synthesis, end repair, A-base addition, and ligation of the Illumina-indexed adaptors were performed according to Illumina’s protocol. Libraries were then size selected for cDNA target fragments of 200-300 bp on 2% Low Range Ultra Agarose followed by PCR amplified using Phusion DNA polymerase (NEB) for 15 PCR cycles. Metatranscriptome sequencing was performed by Shanghai Biozeron Biothchnology Co. , Ltd. (Shanghai, China). All samples were sequenced in the Illumina HiSeq 2500 instrument. Libraries were prepared with a fragment length of approximately 450 bp. Paired-end reads were generated with 150 bp in the forward and reverse directions.

**Reads quality control and mapping.** The raw paired end reads were trimmed and quality controlled by Trimmomatic with parameters (SLIDINGWINDOW:4:15 MINLEN:75) (version 0.36, http://www.usadellab.org/cms/uploads/supplementary/Trimmomatic). This set of high-quality reads was then used for further analysis.

**Metatranscriptome Assembly and Annotation.** The clean reads were aligned to the SILVA SSU (16S/18S) and SILVA LSU (23S/28S) databases in order to remove rRNA related reads using SortMeRNA (http://bioinfo.lifl.fr/RNA/sortmerna/) software (6). Then clean data from all samples were used to do assembly with megahit (v1.0, http://www.l3-bioinfo.com/products/megahit.html). All the genes were predicted by METAProdigal (http://compbio.ornl.gov/prodigal/). Then non-redundant gene catalog were constructed with 95% identity and 90% coverage by CD-HIT (http://www.bioinformatics.org/cd-hit/). All genes searched against the NCBI protein nonredundant (NR, 20180814), String (Version 11.0, https://string-db.org/), and KEGG databases (Release 97.0, http://www.genome.jp/kegg/) using BLASTp to identify the proteins that had the highest sequence similarity with the given transcripts to retrieve their function annotations and a typical cut-off E-values less than 1.0×10^-5^ was set. BLAST2GO (http://www.blast2go.com/b2ghome) program was used to get GO annotations of unique assembled transcripts for describing biological processes, molecular functions and cellular components. Metabolic pathway analysis was performed using the KEGG database.

**Differential expression analysis and functional enrichment.** To identify DEGs (differential expression genes) between the two different samples, the expression level for each transcript was calculated using Salmon (v0.8.0, <https://github.com/COMBINE-lab/salmon>) (7). RSEM (v1.3.3, http://deweylab.biostat.wisc.edu/rsem/) was used to quantify gene and isoform abundances. R statistical package software EdgeR (Empirical analysis of Digital Gene Expression in R, http://www.bioconductor.org/packages/2.12/bioc/html/edgeR.html) was utilized for differential expression analysis. The DEGs between two samples were selected using the following criteria: the logarithmic of fold change was greater than 1 and the false discovery rate (FDR) should be less than 0.05. To understand the functions of the differential expressed genes, GO functional enrichment and KEGG pathway analysis were carried out by Goatools (https://github.com/tanghaibao/Goatools) and KOBAS (Version 3.0, http://kobas.cbi.pku.edu.cn/home.do) respectively. DEGs were significantly enriched in GO terms and metabolic pathways when their Bonferroni-corrected *P-*value was less than 0.05.

**References**

1. Cox J, Mann M. 2008. MaxQuant enables high peptide identification rates, individualized p.p.b.-range mass accuracies and proteome-wide protein quantification. *Nat Biotechnol* 26:1367-72.

2. Kanehisa M, Sato Y, Morishima K. 2016. BlastKOALA and GhostKOALA: KEGG Tools for Functional Characterization of Genome and Metagenome Sequences. *J Mol Biol* 428:726-731.

3. Galperin MY, Wolf YI, Makarova KS, Vera Alvarez R, Landsman D, Koonin EV. 2021. COG database update: focus on microbial diversity, model organisms, and widespread pathogens. *Nucleic Acids Res* 49:D274-D281.

4. Mistry J, Chuguransky S, Williams L, Qureshi M, Salazar GA, Sonnhammer ELL, Tosatto SCE, Paladin L, Raj S, Richardson LJ, Finn RD, Bateman A. 2021. Pfam: The protein families database in 2021. *Nucleic Acids Res* 49:D412-D419.

5. Kanehisa M, Sato Y, Kawashima M, Furumichi M, Tanabe M. 2016. KEGG as a reference resource for gene and protein annotation. *Nucleic Acids Res* 44:D457-D462.

6. Kopylova E, Noé L, Touzet H. 2012. SortMeRNA: fast and accurate filtering of ribosomal RNAs in metatranscriptomic data. *Bioinformatics* 28:3211-7.

7. Patro R, Duggal G, Love MI, Irizarry RA, Kingsford C. 2017. Salmon provides fast and bias-aware quantification of transcript expression. *Nat Methods* 14:417-419.
