# Supplementary material for: Deep-sea in situ and laboratory multi-omics provide insights into the sulfur assimilation of a deep-sea Chloroflexota bacterium
Source: mBio. 2024 Feb 28;15(4):e00004-24. doi: 10.1128/mbio.00004-24 (PMC11005417; doi:10.1128/mbio.00004-24)
Supplement: Table S1 — The genomic analysis of P. methaneseepsis ZRK33. [file mbio.00004-24-s0005.docx]

**Supplementary Table S1.** The genomic analysis of *P*. *methaneseepsis* ZRK33.

| **Gene_id** | **CDS** | | **Size (bp)** | **Gene annotation** |
| --- | --- | --- | --- | --- |
| **Sulfur metabolism** | | | | |
| G4Y79_06955 | 1615948 | 1618197 | 2250 | Sulfite reductase (Sir) |
| G4Y79_10610 | 2479779 | 2480978 | 1200 | Sulfate adenylyltransferase subunit 1 (CysN) |
| G4Y79_01190  G4Y79_06930  G4Y79_06925 | 279133  1611496  1610704 | 280692  1612059  1611357 | 1560  564  654 | Sulfate adenylyltransferase subunit 2 (CysD)  Adenylylsulfate kinase (CysC)  Phosphoadenosine phosphosulfate reductase (CysH) |
| G4Y79_11005 | 2549949 | 2550812 | 864 | Thiosulfate sulfurtransferase (TST) |
| G4Y79_14900 | 3459492 | 3460325 | 834 | Thiosulfate sulfurtransferase (TST) |
| G4Y79_12395 | 2863263 | 2863862 | 600 | Thiosulfate dehydrogenase [quinone] large subunit (DoxD) |
| G4Y79_07260 | 1695224 | 1696156 | 933 | Cysteine synthase (CysK) |
| **EMP Glycolysis** | | | | |
| G4Y79_18225  G4Y79_08170  G4Y79_04220  G4Y79_24425  G4Y79_15765  G4Y79_15760  G4Y79_15575  G4Y79_09005  G4Y79_22845  G4Y79_22840  G4Y79_22835  G4Y79_00130 | 4227956  1891504  956545  5630078  3619089  3617824  3584074  2077948  5299488  5298458  5297202  24733 | 4229374  1894389  957717  5631172  3620096  3619011  3585366  2079447  5300477  5299420  5298458  25749 | 1419  2886  1173  1095  1008  1188  1293  1500  990  963  1257  1017 | Phosphomannomutase/phosphoglucomutase (pmm-pgm)  Glucose-6-phosphate isomerase (GPI)  ATP-dependent phosphofructokinase (pfk)  Fructose-bisphosphate aldolase  Glyceraldehyde 3-phosphate dehydrogenase  Phosphoglycerate kinase (PGK)  Enolase  Pyruvate kinase (pyk)  Pyruvate dehydrogenase E1 component alpha subunit (PdhA)  Pyruvate dehydrogenase E1 component beta subunit (PdhB)  Pyruvate dehydrogenase E2 component (PdhC)  Glyceraldehyde-3-phosphate dehydrogenase |
| **Oxidative Pentose Phosphate Pathway** | | | | |
| G4Y79_06540  G4Y79_08660  G4Y79_12570  G4Y79_08175  G4Y79_19585  G4Y79_06820 | 1517062  1999496  2905731  1894838  4543084  1583958 | 1518150  2001541  2906522  1895302  4544118  1584932 | 1089  2046  792  465  1035  975 | Transaldolase  Transketolase  Ribulose-phosphate 3-epimerase  Ribose 5-phosphate isomerase B (RpiB)  Ribose-phosphate pyrophosphokinase  Deoxyribose-phosphate aldolase (DeoC) |
| **TCA cycle** | | | | |
| G4Y79_03575  G4Y79_12695  G4Y79_08390  G4Y79_16970  G4Y79_16975  G4Y79_20290  G4Y79_12650  G4Y79_12655  G4Y79_23720  G4Y79_23725  G4Y79_10075  G4Y79_10080  G4Y79_03915  G4Y79_03920  G4Y79_17370  G4Y79_17955 | 816034  2934060  1943872  3925011  3926281  4720178  2925304  2926348  5475519  5476398  2348343  2350192  890147  892094  4023834  4154173 | 817290  2936771  1945323  3926264  3929076  4721578  2926341  2928192  5476397  5477534  2350145  2350992  891985  892846  4025234  4155159 | 1257  2712  1452  1254  2796  1401  1038  1845  879  1137  1803  801  1839  753  1401  987 | Citrate synthase  Aconitate hydratase  Isocitrate dehydrogenase  2-oxoglutarate dehydrogenase E2 component (sucB)  2-oxoglutarate dehydrogenase E1 component (sucA)  Dihydrolipoyl dehydrogenase  2-oxoglutarate/2-oxoacid ferredoxin oxidoreductase subunit beta (oforB)  2-oxoglutarate/2-oxoacid ferredoxin oxidoreductase subunit beta (oforA)  Succinyl-CoA synthetase alpha subunit (sucD)  Succinyl-CoA synthetase beta subunit (sucC)  Succinate dehydrogenase (ubiquinone) flavoprotein subunit (sdhA)  Succinate dehydrogenase (ubiquinone) iron-sulfur subunit (sdhB)  Succinate dehydrogenase flavoprotein subunit (frdA)  Succinate dehydrogenase iron-sulfur subunit (frdB)  Fumarate hydratase  Malate dehydrogenase |
| **Urea cycle** | | | | |
| G4Y79_19965  G4Y79_17675  G4Y79_18985  G4Y79_14920  G4Y79_14925 | 4644885  4088900  4385770  3463630  3465016 | 4645676  4089880  4386687  3464895  3466398 | 792  981  918  1266  1383 | Arginase  Ornithine carbamoyltransferase  Ornithine carbamoyltransferase  Argininosuccinate synthase  Argininosuccinate lyase |
| **Others** | | | | |
| G4Y79_17500  G4Y79_17485  G4Y79_17490  G4Y79_17495  G4Y79_19740  G4Y79_19745  G4Y79_09515  G4Y79_09510  G4Y79_10380  G4Y79_10385  G4Y79_10390  G4Y79_10395  G4Y79_10400  G4Y79_10405  G4Y79_10415  G4Y79_10420  G4Y79_10425  G4Y79_10430  G4Y79_10435  G4Y79_10440  G4Y79_10445  G4Y79_10450  G4Y79_19760  G4Y79_19765  G4Y79_19770  G4Y79_19775  G4Y79_19780 | 4051977  4048697  4049191  4050626  4582343  4583187  2203407  2202375  2428025  2428375  2428960  2429565  2430869  2431399  2433199  2435822  2437383  2437990  2439476  2439847  2442515  2444207  4585712  4587257  4588983  4590687  4593120 | 4053479  4049155  4050594  4051849  4583164  4583420  2204825  2203394  2428384  2428860  2429532  2430827  2431372  2432688  2435748  2437297  2437877  2439438  2439787  2442498  2444173  2445763  4587256  4588978  4590677  4593110  4593455 | 1503  459  1404  1224  822  234  1419  1020  360  486  573  1263  504  1290  2550  1476  495  1449  312  2652  1659  1557  1545  1722  1695  2424  336 | F-type H+-transporting ATPase subunit alpha (atpA)  F-type H+-transporting ATPase subunit alpha (atpC)  F-type H+-transporting ATPase subunit alpha (atpD)  F-type H+-transporting ATPase subunit alpha (atpG)  F-type H+-transporting ATPase subunit alpha (atpF)  F-type H+-transporting ATPase subunit alpha (atpE)  Cytochrome bd ubiquinol oxidase subunit I (cydA)  Cytochrome bd ubiquinol oxidase subunit I (cydB)  NADH-quinone oxidoreductase subunit A (nuoA)  NADH-quinone oxidoreductase subunit A (nuoB)  NADH-quinone oxidoreductase subunit A (nuoC)  NADH-quinone oxidoreductase subunit A (nuoD)  NADH-quinone oxidoreductase subunit A (nuoE)  NADH-quinone oxidoreductase subunit A (nuoF)  NADH-quinone oxidoreductase subunit A (nuoG)  NADH-quinone oxidoreductase subunit A (nuoH)  NADH-quinone oxidoreductase subunit A (nuoI)  NADH-quinone oxidoreductase subunit A (nuoJ)  NADH-quinone oxidoreductase subunit A (nuoK)  NADH-quinone oxidoreductase subunit A (nuoL)  NADH-quinone oxidoreductase subunit A (nuoM)  NADH-quinone oxidoreductase subunit A (nuoN)  NADH-quinone oxidoreductase subunit A (nuoN)  NADH-quinone oxidoreductase subunit A (nuoM)  NADH-quinone oxidoreductase subunit A (nuoM)  NADH-quinone oxidoreductase subunit A (nuoL)  NADH-quinone oxidoreductase subunit A (nuoK) |
