# Supplementary material for: Deep-sea in situ and laboratory multi-omics provide insights into the sulfur assimilation of a deep-sea Chloroflexota bacterium
Source: mBio. 2024 Feb 28;15(4):e00004-24. doi: 10.1128/mbio.00004-24 (PMC11005417; doi:10.1128/mbio.00004-24)
Supplement: Table S2 — The proteomic analysis of P. methaneseepsis ZRK33 cultured in rich medium alone or supplemented with either Na2SO4 or Na2S2O3. [file mbio.00004-24-s0006.docx]

**Supplementary Table S2.** The proteomic analysis of *P*. *methaneseepsis* ZRK33 cultured in rich medium alone or supplemented with either Na_2_SO_4_ or Na_2_S_2_O_3_. “None” indicates that no corresponding protein is detected.

| **Gene_id** | **Sulfate/Control Ratio** | **Thiosulfate /Control Ratio** | | ***P*-value** | **Protein** |
| --- | --- | --- | --- | --- | --- |
| **Sulfur metabolism** | | | | | |
| G4Y79_06955 | 0.904 | 0.739 | 0.0051838 | | Sulfite reductase (Sir) |
| G4Y79_10610 | 1.519 | 0.747 | 4.4398E-26 | | Sulfate adenylyltransferase subunit 1 (CysN) |
| G4Y79_01190  G4Y79_06930  G4Y79_06925 | 0.778  None  None | 1.387  None  None | 0.00031265  None  None | | Sulfate adenylyltransferase subunit 2 (CysD)  Adenylylsulfate kinase (CysC)  Phosphoadenosine phosphosulfate reductase (CysH) |
| G4Y79_11005 | 1.425 | 1.322 | 0.0000044141 | | Thiosulfate sulfurtransferase (TST) |
| G4Y79_14900 | 1.729 | 2.617 | 0.000086128 | | Thiosulfate sulfurtransferase (TST) |
| G4Y79_12395 | None | None | None | | Thiosulfate dehydrogenase [quinone] large subunit (DoxD) |
| G4Y79_07260 | None | None | None | | Cysteine synthase (CysK) |
| **EMP Glycolysis** | | | | | |
| G4Y79_18225  G4Y79_08170  G4Y79_04220  G4Y79_24425  G4Y79_15765  G4Y79_15760  G4Y79_15575  G4Y79_09005  G4Y79_22845  G4Y79_22840  G4Y79_22835  G4Y79_00130 | 1.353  1.382  1.214  0.968  1.41  1.165  0.702  1.294  0.784  0.768  0.662  1.624 | 1.119  0.972  1.378  1.814  1.648  1.065  0.749  1.236  0.991  0.819  1.004  2.144 | 0.00000000000065839  0.000015396  0.014254  0.0058994  0.0000000000000024918  0.0000000036738  0.00056157  0.00000000000014439  0.00001897  0.01605  4.3156E-19  4.2385E-25 | | Phosphomannomutase/phosphoglucomutase (pmm-pgm)  Glucose-6-phosphate isomerase (GPI)  ATP-dependent phosphofructokinase (pfk)  Fructose-bisphosphate aldolase  Glyceraldehyde 3-phosphate dehydrogenase  Phosphoglycerate kinase (PGK)  Enolase  Pyruvate kinase (pyk)  Pyruvate dehydrogenase E1 component alpha subunit (PdhA)  Pyruvate dehydrogenase E1 component beta subunit (PdhB)  Pyruvate dehydrogenase E2 component (PdhC)  Glyceraldehyde-3-phosphate dehydrogenase |
| **Oxidative Pentose Phosphate Pathway** | | | | | |
| G4Y79_06540  G4Y79_08660  G4Y79_12570  G4Y79_08175  G4Y79_19585  G4Y79_06820 | 0.851  0.97  None  0.568  3.747  None | 0.859  1.135  None  1.01  1.28  None | 0.0086396  3.4417E-42  None  0.00013953  0.010803  None | | Transaldolase  Transketolase  Ribulose-phosphate 3-epimerase  Ribose 5-phosphate isomerase B (RpiB)  Ribose-phosphate pyrophosphokinase  Deoxyribose-phosphate aldolase (DeoC) |
| **TCA cycle** | | | | | |
| G4Y79_03575  G4Y79_12695  G4Y79_08390  G4Y79_16970  G4Y79_16975  G4Y79_20290  G4Y79_12650  G4Y79_12655  G4Y79_23720  G4Y79_23725  G4Y79_10075  G4Y79_10080  G4Y79_03915  G4Y79_03920  G4Y79_17370  G4Y79_17955 | None  0.961  1.138  0.801  1.013  1.735  0.622  1.103  0.883  1.124  1.121  1.248  1.216  0.66  1.513  2.496 | None  0.885  0.999  0.825  1.235  1.978  1.107  1.765  1.091  0.626  1.14  0.679  1.382  0.584  1.242  1.752 | None  1.556E-42  0.038229  0.0000000032559  0.00028834  0.00000000013835  0.000000011219  0.000021821  0.00000061937  0.00016122  0.00019897  0.0000000073315  0.0000024459  0.0019718  0.000079075  0.0000000000000094546 | | Citrate synthase  Aconitate hydratase  Isocitrate dehydrogenase  2-oxoglutarate dehydrogenase E2 component (sucB)  2-oxoglutarate dehydrogenase E1 component (sucA)  Dihydrolipoyl dehydrogenase  2-oxoglutarate/2-oxoacid ferredoxin oxidoreductase subunit beta (oforB)  2-oxoglutarate/2-oxoacid ferredoxin oxidoreductase subunit beta (oforA)  Succinyl-CoA synthetase alpha subunit (sucD)  Succinyl-CoA synthetase beta subunit (sucC)  Succinate dehydrogenase (ubiquinone) flavoprotein subunit (sdhA)  Succinate dehydrogenase (ubiquinone) iron-sulfur subunit (sdhB)  Succinate dehydrogenase flavoprotein subunit (frdA)  Succinate dehydrogenase iron-sulfur subunit (frdB)  Fumarate hydratase  Malate dehydrogenase |
| **Urea cycle** | | | | | |
| G4Y79_19965  G4Y79_17675  G4Y79_18985  G4Y79_14920  G4Y79_14925 | None  1.469  0.989  1.212  0.789 | None  1.119  0.863  1.443  1.422 | None  0.0040294  0.013854  1.5716E-24  0.000029069 | | Arginase  Ornithine carbamoyltransferase  Ornithine carbamoyltransferase  Argininosuccinate synthase  Argininosuccinate lyase |
| **Others** | | | | | |
| G4Y79_17500  G4Y79_17485  G4Y79_17490  G4Y79_17495  G4Y79_19740  G4Y79_19745  G4Y79_09515  G4Y79_09510  G4Y79_10380  G4Y79_10385  G4Y79_10390  G4Y79_10395  G4Y79_10400  G4Y79_10405  G4Y79_10415  G4Y79_10420  G4Y79_10425  G4Y79_10430  G4Y79_10435  G4Y79_10440  G4Y79_10445  G4Y79_10450  G4Y79_19760  G4Y79_19765  G4Y79_19770  G4Y79_19775  G4Y79_19780 | 1.032  0.764  1.701  2.818  1.108  1.288  3.326  None  3.628  0.269  1.286  1.986  1.845  0.886  1.033  None  1.011  None  None  None  None  5.119  None  None  None  1.894  None | 1.029  0.395  1.042  2.306  0.801  1.219  1.748  None  1.457  1.002  0.49  1.337  0.587  0.82  1.002  None  1.178  None  None  None  None  1.283  None  None  None  7.768  None | 0.0042358  0.00032167  0.002893  0.0057179  0.006393  0.00057384  0.0073807  None  0.0000000006835  0.006846  0.005378  0.0039472  0.00000026423  0.00000000000018414  0.0000000041215  None  0.0045018  None  None  None  None  0.0023223  None  None  None  2.9548E-18  None | | F-type H+-transporting ATPase subunit alpha (atpA)  F-type H+-transporting ATPase subunit alpha (atpC)  F-type H+-transporting ATPase subunit alpha (atpD)  F-type H+-transporting ATPase subunit alpha (atpG)  F-type H+-transporting ATPase subunit alpha (atpF)  F-type H+-transporting ATPase subunit alpha (atpE)  Cytochrome bd ubiquinol oxidase subunit I (cydA)  Cytochrome bd ubiquinol oxidase subunit I (cydB)  NADH-quinone oxidoreductase subunit A (nuoA)  NADH-quinone oxidoreductase subunit A (nuoB)  NADH-quinone oxidoreductase subunit A (nuoC)  NADH-quinone oxidoreductase subunit A (nuoD)  NADH-quinone oxidoreductase subunit A (nuoE)  NADH-quinone oxidoreductase subunit A (nuoF)  NADH-quinone oxidoreductase subunit A (nuoG)  NADH-quinone oxidoreductase subunit A (nuoH)  NADH-quinone oxidoreductase subunit A (nuoI)  NADH-quinone oxidoreductase subunit A (nuoJ)  NADH-quinone oxidoreductase subunit A (nuoK)  NADH-quinone oxidoreductase subunit A (nuoL)  NADH-quinone oxidoreductase subunit A (nuoM)  NADH-quinone oxidoreductase subunit A (nuoN)  NADH-quinone oxidoreductase subunit A (nuoN)  NADH-quinone oxidoreductase subunit A (nuoM)  NADH-quinone oxidoreductase subunit A (nuoM)  NADH-quinone oxidoreductase subunit A (nuoL)  NADH-quinone oxidoreductase subunit A (nuoK) |
