# Supplementary material for: Deep-sea in situ and laboratory multi-omics provide insights into the sulfur assimilation of a deep-sea Chloroflexota bacterium
Source: mBio. 2024 Feb 28;15(4):e00004-24. doi: 10.1128/mbio.00004-24 (PMC11005417; doi:10.1128/mbio.00004-24)
Supplement: Table S3 — In situ transcriptomic analysis of P. methaneseepsis ZRK33 cultured in deep-sea conditions. [file mbio.00004-24-s0007.docx]

**Supplementary Table S3.** *In situ* transcriptomic analysis of *P*. *methaneseepsis* ZRK33 cultured in deep-sea conditions.

| **Gene_id** | **Log_2_ fold change** | ***P*-value** | **Gene annotation** |
| --- | --- | --- | --- |
| **Sulfur metabolism** | | | |
| G4Y79_06955 | 1.633198009  1.343688999  2.031627715  0.21320173  0.897271817  2.387446648  -1.434626953  0.90558333  0.464726489 | 0.000632164 | Sulfite reductase (Sir) |
| G4Y79_10610  G4Y79_01190 |  | 0.00134895  0.00000335 | Sulfate adenylyltransferase subunit 1 (CysN)  Sulfate adenylyltransferase subunit 2 (CysD) |
| G4Y79_06930  G4Y79_06925 |  | 0.886004739  0.289175799 | Adenylylsulfate kinase (CysC)  Phosphoadenosine phosphosulfate reductase (CysH) |
| G4Y79_11005 |  | 0.00000018 | Thiosulfate sulfurtransferase (TST) |
| G4Y79_14900 |  | 0.000884874 | Thiosulfate sulfurtransferase (TST) |
| G4Y79_12395 |  | 0.027413195 | Thiosulfate dehydrogenase [quinone] large subunit (DoxD) |
| G4Y79_07260 |  | 0.282000488 | Cysteine synthase (CysK) |
| **EMP Glycolysis** | | | |
| G4Y79_18225  G4Y79_08170  G4Y79_04220  G4Y79_24425  G4Y79_15765  G4Y79_15760  G4Y79_15575  G4Y79_09005  G4Y79_22845  G4Y79_22840  G4Y79_22835  G4Y79_00130 | -0.661451683  1.598447765  -2.100302941  -0.422055497  -1.730310315  1.294743885  0.442131839  -1.342467035  -0.728658715  -0.704281971  -0.828266197  -1.625336874 | 0.12532854  0.000157218  0.0000069  0.310772872  0.0000646  0.003693541  0.304316066  0.003457928  0.086026477  0.093243189  0.047140704  0.000129673 | Phosphomannomutase/phosphoglucomutase (pmm-pgm)  Glucose-6-phosphate isomerase (GPI)  ATP-dependent phosphofructokinase (pfk)  Fructose-bisphosphate aldolase  Glyceraldehyde 3-phosphate dehydrogenase  Phosphoglycerate kinase (PGK)  Enolase  Pyruvate kinase (pyk)  Pyruvate dehydrogenase E1 component alpha subunit (PdhA)  Pyruvate dehydrogenase E1 component beta subunit (PdhB)  Pyruvate dehydrogenase E2 component (PdhC)  Glyceraldehyde-3-phosphate dehydrogenase |
| **Oxidative Pentose Phosphate Pathway** | | | |
| G4Y79_06540  G4Y79_08660  G4Y79_12570  G4Y79_08175  G4Y79_19585  G4Y79_06820 | -1.285173949  -0.152742898  0.116522899  2.732030927  0.105275254  -1.842373429 | 0.00210091  0.715282036  0.791177137  0.000000000995  0.813572947  0.000018 | Transaldolase  Transketolase  Ribulose-phosphate 3-epimerase  Ribose 5-phosphate isomerase B (RpiB)  Ribose-phosphate pyrophosphokinase  Deoxyribose-phosphate aldolase (DeoC) |
| **TCA cycle** | | | |
| G4Y79_03575  G4Y79_12695  G4Y79_08390  G4Y79_16970  G4Y79_16975  G4Y79_20290  G4Y79_12650  G4Y79_12655  G4Y79_23720  G4Y79_23725  G4Y79_10075  G4Y79_10080  G4Y79_03915  G4Y79_03920  G4Y79_17370  G4Y79_17955 | -1.402857583  -0.398876299  -0.212589135  -0.331242963  -0.842963369  -1.076139838  0.963821302  0.508941064  0.324980649  0.092008005  -0.952593075  -0.594830131  -2.170671947  -2.003497779  -0.682336083  0.627317752 | 0.00083979  0.333702406  0.626344417  0.443277008  0.04680403  0.009772245  0.046428624  0.253869603  0.447071263  0.841071252  0.055612972  0.283815604  0.000000441  0.0000038  0.105699307  0.138848381 | Citrate synthase  Aconitate hydratase  Isocitrate dehydrogenase  2-oxoglutarate dehydrogenase E2 component (sucB)  2-oxoglutarate dehydrogenase E1 component (sucA)  Dihydrolipoyl dehydrogenase  2-oxoglutarate/2-oxoacid ferredoxin oxidoreductase subunit beta (oforB)  2-oxoglutarate/2-oxoacid ferredoxin oxidoreductase subunit beta (oforA)  Succinyl-CoA synthetase alpha subunit (sucD)  Succinyl-CoA synthetase beta subunit (sucC)  Succinate dehydrogenase (ubiquinone) flavoprotein subunit (sdhA)  Succinate dehydrogenase (ubiquinone) iron-sulfur subunit (sdhB)  Succinate dehydrogenase flavoprotein subunit (frdA)  Succinate dehydrogenase iron-sulfur subunit (frdB)  Fumarate hydratase  Malate dehydrogenase |
| **Urea cycle** | | | |
| G4Y79_19965  G4Y79_17675  G4Y79_18985  G4Y79_14920  G4Y79_14925 | 1.816735722  0.651215655  -0.679264444  2.637675399  3.998516587 | 0.000192095  0.515477243  0.128372401  0.000000246  0.00000401 | Arginase  Ornithine carbamoyltransferase  Ornithine carbamoyltransferase  Argininosuccinate synthase  Argininosuccinate lyase |
| **Others** | | | |
| G4Y79_17500  G4Y79_17485  G4Y79_17490  G4Y79_17495  G4Y79_19740  G4Y79_19745  G4Y79_09515  G4Y79_09510  G4Y79_10380  G4Y79_10385  G4Y79_10390  G4Y79_10395  G4Y79_10400  G4Y79_10405  G4Y79_10415  G4Y79_10420  G4Y79_10425  G4Y79_10430  G4Y79_10435  G4Y79_10440  G4Y79_10445  G4Y79_10450  G4Y79_19760  G4Y79_19765  G4Y79_19770  G4Y79_19775  G4Y79_19780 | 0.030613574  1.108044774  1.218193346  1.232247177  0.221702065  0.22890855  -0.455400079  0.351746767  -1.861385457  -1.3758423  -0.874611581  -0.261766625  0.329127579  0.225485367  -0.114471905  -0.50663684  -0.499687804  0.500200992  0.042938229  0.80412192  1.303437996  1.377354018  2.281558967  2.063588548  1.787365747  1.423243815  1.547492236 | 0.943449141  0.01117121  0.003977843  0.003947159  0.595738834  0.62055419  0.296681018  0.449192073  0.0000338  0.00122848  0.037826692  0.534051714  0.494989666  0.596078198  0.785477237  0.229757581  0.267948431  0.247951664  0.957625675  0.059228822  0.002605022  0.002367795  0.000000995  0.0000106  0.0000848  0.001020022  0.004698802 | F-type H+-transporting ATPase subunit alpha (atpA)  F-type H+-transporting ATPase subunit alpha (atpC)  F-type H+-transporting ATPase subunit alpha (atpD)  F-type H+-transporting ATPase subunit alpha (atpG)  F-type H+-transporting ATPase subunit alpha (atpF)  F-type H+-transporting ATPase subunit alpha (atpE)  Cytochrome bd ubiquinol oxidase subunit I (cydA)  Cytochrome bd ubiquinol oxidase subunit I (cydB)  NADH-quinone oxidoreductase subunit A (nuoA)  NADH-quinone oxidoreductase subunit A (nuoB)  NADH-quinone oxidoreductase subunit A (nuoC)  NADH-quinone oxidoreductase subunit A (nuoD)  NADH-quinone oxidoreductase subunit A (nuoE)  NADH-quinone oxidoreductase subunit A (nuoF)  NADH-quinone oxidoreductase subunit A (nuoG)  NADH-quinone oxidoreductase subunit A (nuoH)  NADH-quinone oxidoreductase subunit A (nuoI)  NADH-quinone oxidoreductase subunit A (nuoJ)  NADH-quinone oxidoreductase subunit A (nuoK)  NADH-quinone oxidoreductase subunit A (nuoL)  NADH-quinone oxidoreductase subunit A (nuoM)  NADH-quinone oxidoreductase subunit A (nuoN)  NADH-quinone oxidoreductase subunit A (nuoN)  NADH-quinone oxidoreductase subunit A (nuoM)  NADH-quinone oxidoreductase subunit A (nuoM)  NADH-quinone oxidoreductase subunit A (nuoL)  NADH-quinone oxidoreductase subunit A (nuoK) |
