# Supplementary material for: Deep-sea in situ and laboratory multi-omics provide insights into the sulfur assimilation of a deep-sea Chloroflexota bacterium
Source: mBio. 2024 Feb 28;15(4):e00004-24. doi: 10.1128/mbio.00004-24 (PMC11005417; doi:10.1128/mbio.00004-24)
Supplement: Table S4 — Assembly statistics and quality metrics of reconstructed genome bins of Chloroflexota used in this study. [file mbio.00004-24-s0008.docx]

**Supplementary Table S4.** Assembly statistics and quality metrics of reconstructed genome bins of *Chloroflexota* used in this study.

| **Bin name** | **Taxonomy** | **Completeness (%)** | **Contamination (%)** | **GC (%)** | **N50 (bp)** | **Genome size (bp)** |
| --- | --- | --- | --- | --- | --- | --- |
| zhu.bin.33 | *Chloroflexota* | 78.05 | 1.99 | 61.3 | 3481 | 2239783 |
| zhu.bin.3 | *Chloroflexota* | 56.84 | 1.98 | 0.494 | 4097 | 748332 |
| zhu.bin.7 | *Chloroflexota* | 59.57 | 1.98 | 0.446 | 5817 | 588020 |
| zhu.bin.9 | *Chloroflexota* | 66.38 | 1.925 | 0.506 | 2239 | 1167066 |
| zhu.bin.22 | *Chloroflexota* | 51.94 | 8.91 | 0.52 | 6548 | 1409231 |
| zhu.bin.44 | *Chloroflexota* | 66.88 | 0.99 | 0.528 | 6754 | 946329 |
| C1.bin.34 | *Chloroflexota* | 76.21 | 2.828 | 0.612 | 3548 | 2588152 |
| C1.bin.35 | *Chloroflexota* | 58.64 | 1.818 | 0.455 | 8245 | 1933721 |
| C2.bin.4 | *Chloroflexota* | 82.83 | 0 | 0.486 | 39431 | 941411 |
| C2.bin.6 | *Chloroflexota* | 70.92 | 0 | 0.495 | 7628 | 827319 |
| C2.bin.8 | *Chloroflexota* | 74.02 | 0.99 | 0.525 | 4817 | 757107 |
| C2.bin.9 | *Chloroflexota* | 80.36 | 1.98 | 0.548 | 6764 | 1051572 |
| C2.bin.12 | *Chloroflexota* | 54.49 | 2.727 | 0.523 | 3759 | 1643107 |
| C2.bin.17 | *Chloroflexota* | 65.4 | 4.158 | 0.542 | 4882 | 621181 |
| C2.bin.33 | *Chloroflexota* | 63.82 | 1.386 | 0.609 | 3494 | 1094429 |
| C2.bin.34 | *Chloroflexota* | 62.68 | 2.727 | 0.479 | 4652 | 2209326 |
| C2.bin.38 | *Chloroflexota* | 72.49 | 4.022 | 0.619 | 3598 | 2727830 |
| C2.bin.45 | *Chloroflexota* | 87.29 | 1.485 | 0.537 | 9527 | 1647588 |
| C2.bin.48 | *Chloroflexota* | 61.22 | 8.25 | 0.452 | 5264 | 973927 |
| C4.bin.19 | *Chloroflexota* | 67.43 | 0.565 | 0.644 | 3026 | 1749317 |
| H1.bin.7 | *Chloroflexota* | 73.68 | 0 | 0.545 | 4996 | 1405141 |
| H1.bin.32 | *Chloroflexota* | 71.94 | 7.727 | 0.563 | 3845 | 2766581 |
| H2.bin.45 | *Chloroflexota* | 76.73 | 4.378 | 0.579 | 3649 | 942250 |
| H2.bin.80 | *Chloroflexota* | 86.57 | 0.925 | 0.663 | 14655 | 3235763 |
| H2.bin.87 | *Chloroflexota* | 59.82 | 1.485 | 0.603 | 3209 | 1355795 |
| H2.bin.116 | *Chloroflexota* | 92.73 | 0.99 | 0.543 | 27621 | 1910274 |
| H2.bin.125 | *Chloroflexota* | 70.13 | 0.99 | 0.477 | 5832 | 1871708 |
